# Extent of myosin penetration within the actin cortex regulates cell surface mechanics

**DOI:** 10.1038/s41467-021-26611-2

**Published:** 2021-11-11

**Authors:** Binh An Truong Quang, Ruby Peters, Davide A. D. Cassani, Priyamvada Chugh, Andrew G. Clark, Meghan Agnew, Guillaume Charras, Ewa K. Paluch

**Affiliations:** 1 MRC Laboratory for Molecular Cell Biology, University College London, London, WC1E 6BT UK; 2 Department of Physiology, Development and Neuroscience, University of Cambridge, Cambridge, CB2 3DY UK; 3 London Centre for Nanotechnology, University College London, London, WC1H 0AH UK; 4 Department of Cell and Developmental Biology, University College London, London, WC1E 6BT UK; 5 Present Address: University of Stuttgart, Institute of Cell Biology and Immunology, Allmandring 31, 70569 Stuttgart, Germany

**Keywords:** Biophysics, Super-resolution microscopy, Actin, Myosin

## Abstract

In animal cells, shape is mostly determined by the actomyosin cortex, a thin cytoskeletal network underlying the plasma membrane. Myosin motors generate tension in the cortex, and tension gradients result in cellular deformations. As such, many cell morphogenesis studies have focused on the mechanisms controlling myosin activity and recruitment to the cortex. Here, we demonstrate using super-resolution microscopy that myosin does not always overlap with actin at the cortex, but remains restricted towards the cytoplasm in cells with low cortex tension. We propose that this restricted penetration results from steric hindrance, as myosin minifilaments are considerably larger than the cortical actin meshsize. We identify myosin activity and actin network architecture as key regulators of myosin penetration into the cortex, and show that increasing myosin penetration increases cortical tension. Our study reveals that the spatial coordination of myosin and actin at the cortex regulates cell surface mechanics, and unveils an important mechanism whereby myosin size controls its action by limiting minifilament penetration into the cortical actin network. More generally, our findings suggest that protein size could regulate function in dense cytoskeletal structures.

## Abstract (web summary)

Cellular deformations are largely driven by contractile forces generated by myosin motors in the submembraneous actin cortex. Here we show that these forces are controlled not simply by cortical myosin levels, but rather by myosins spatial arrangement, specifically the extent of their overlap with cortical actin.

## Introduction

Precise regulation of cellular shape is essential for developmental morphogenesis and tissue homeostasis, and cell shape deregulation has been implicated in diverse diseases, including cancer^1,2^. Animal cell shape is predominantly controlled by the actomyosin cortex, a thin (~150 nm thick) cytoskeletal network that underlies the plasma membrane^3,4^. Myosin motors generate tension in the cortex, and tension gradients drive cellular deformations^5,6^. To understand how cell shape is controlled, it is thus key to elucidate the regulation of cortical tension.

Cortical tension has long been thought to be primarily controlled by the concentration and activity of myosin-II motors at the cortex^7–9^. Yet, recent work has highlighted that the spatial organization of the cortical network is also a key determinant of tension (reviewed in Ref. ^10^). Specifically, work in vitro and in vivo has shown that the length, connectivity, and spatial arrangement of cortical actin filaments can, alongside myosin activity, modulate cortex tension^11–13^. As myosin motors generate contractile forces in the cortex, it is likely that their spatial arrangement within the cortical actin network would also affect tension generation^10^. Recent studies have resolved individual myosin minifilaments in structures like the cytokinetic contractile ring, the lamellum, and in *Drosophila* epithelia, by high-resolution microscopy^14–17^. However, the nanoscale organization of myosins at the cell cortex remains poorly understood, and to what extent changes in myosin organization affect cortical tension is not known.

Here, we use super-resolution microscopy to interrogate the spatial organization of myosin motors at the cortex. We compare cortices of mitotic and rounded interphase HeLa cells, since it is well established that cortical tension increases over threefold between these two cell cycle phases^7,12^. Using Structured Illumination Microscopy (SIM), we first assess myosin minifilament orientation at the cortex and conclude that changes in orientation alone cannot account for the mitotic tension increase. Using two-color direct–Stochastic Optical Reconstruction Microscopy (dSTORM), we then find that in cells with low cortex tension, myosin does not fully overlap with the actin cortex. We demonstrate that changing myosin activity or actin network architecture modulates cortical myosin penetration, and that cortical tension correlates with the level of actin-myosin overlap. Together, our data identifies myosin penetration into the cortex as an important regulatory mechanism controlling cortical tension.

## Results

### Changes in the amount, activity, or orientation of cortical myosin motors are unlikely to account for the cortical tension increase between interphase and mitosis

We first asked whether changes in the amount or activity of cortical myosin could be sufficient to account for the strong increase in cortex tension observed between rounded interphase and mitotic cells. For this, we compared cells synchronized with thymidine, to arrest the majority of cells in G1/S phase (interphase), with cells synchronized with S-trityl-L-cysteine (STLC), to obtain a cell population enriched in prometaphase (mitotic, Supplementary Fig. 1a; see “Methods”). We quantified the intensity of myosin at the cortex using antibodies against myosin-regulatory light chain (MRLC), and myosin heavy chains IIA (MYH9) and IIB (MYH10) (Supplementary Fig. 1b–e). Surprisingly, while cortical MYH10 levels were slightly higher in mitosis, the mean intensities of cortical MRLC and MYH9 were in fact lower in mitosis compared with interphase cells (Supplementary Fig. 1d, e). We further quantified the levels of phosphorylated MRLC, since MRLC phosphorylation enhances myosin activity^18^. Even though MRLC was overall more phosphorylated in mitotic than in interphase cells (Supplementary Fig. 1f, g), immunostainings comparing cortical levels between interphase and mitosis revealed no significant change for biphosphorylated MRLC and a decrease in mitosis for monophosphorylated MRLC (Supplementary Fig. 1d, e). Taken together, these observations suggest that the increase in cortical tension between interphase and mitosis is not likely due to increased cortical myosin-II amounts and activity alone.

**Fig. 1:**
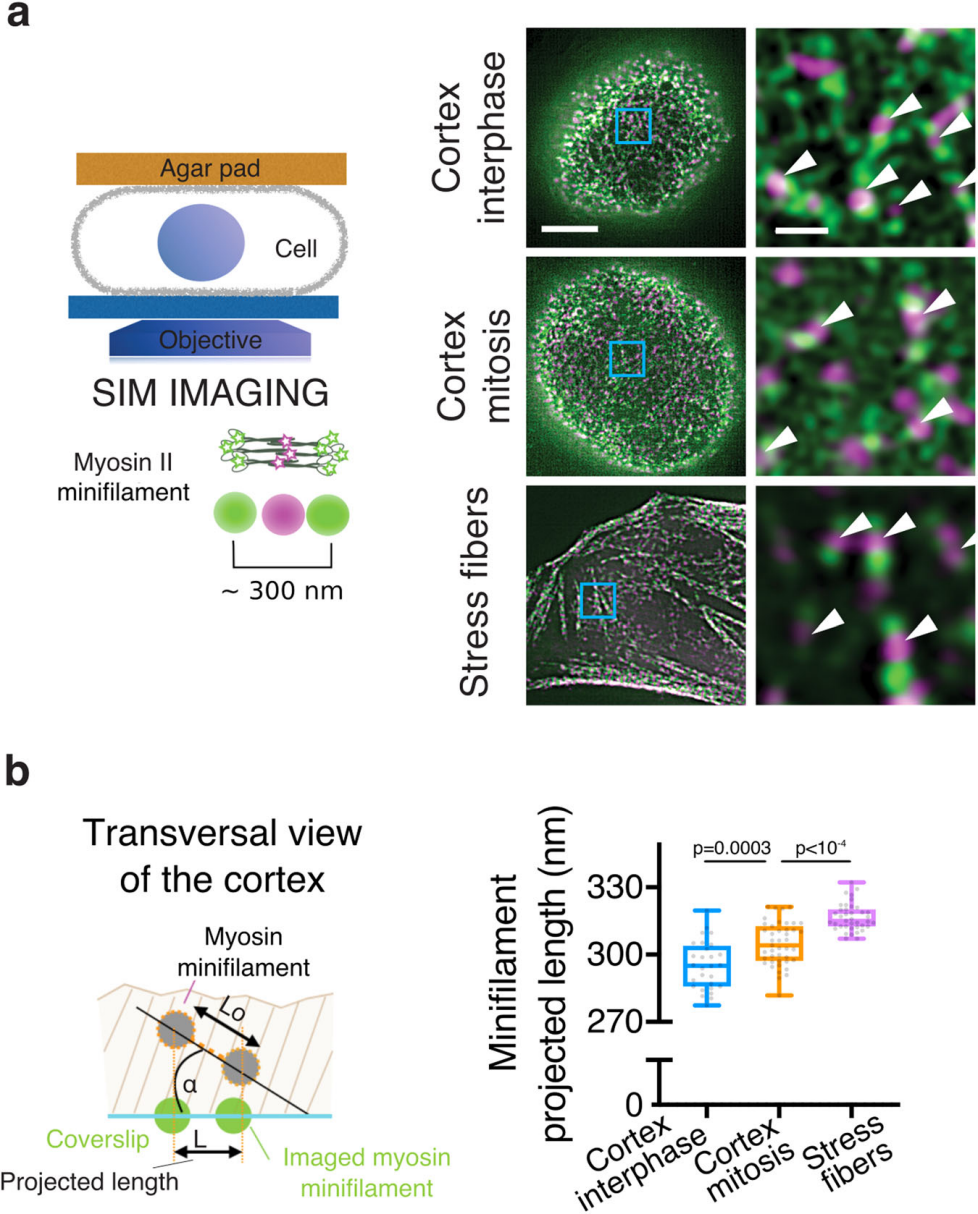
Changes in myosin minifilament orientation between interphase and mitosis. **a** Left: schematic representation of the SIM imaging setup. Cells are compressed under an agar pad, so that a large flat cortical surface can be imaged. Right: representative SIM images of the bottom surface of rounded interphase, mitotic, and spread interphase cells expressing EGFP–NMM HC IIB and NMM HC IIB-tdTom. Arrowheads highlight myosin minifilaments. Scale bars, 5 µm and 0.5 µm. **b** Left: schematic of the minifilament projected length measurement (see Supplementary Fig. 2 for method). Right: projected lengths of myosin minifilaments in the cortex of rounded interphase and mitotic cells, and in stress fibers in spread interphase cells; *n* = 28, 43, and 51 cells, and 2, 3, and 2 independent experiments, respectively; 100–500 minifilaments measured per cell. Boxplots show 25th–75th percentiles, the median, and whiskers from minimum to maximum. Statistics: Welch’s *t-*test (two-tailed). Source data are provided as a Source Data file.

We next examined the spatial arrangement of myosin motors in the interphase and mitotic cortex by super-resolution microscopy. Nonmuscle myosin (NMM) II assembles into ~300 nm-long bipolar minifilaments that exert forces on actin filaments of opposing polarities^19^. We thus explored the spatial orientation of myosin minifilaments in the actomyosin cortex at high spatial resolution using SIM. We coexpressed EGFP-NMM heavy-chain (HC) IIB and NMM HC IIB-tdTom to label myosin II heads and tails, respectively, such that an individual minifilament would appear as two EGFP dots separated by a tdTom dot^20^ (Fig. 1a). We confined cells under an agar pad so that a large portion of the cortex was visible in the SIM imaging plane (schematic in Fig. 1a), and found that individual myosin minifilaments could be visualized at the cortex of interphase and mitotic live cells (Fig. 1a). We next assessed myosin orientation in the cortex by measuring the projected lengths of cortical minifilaments (Fig. 1a and Supplementary Fig. 2). Minifilament projected lengths increased slightly between interphase and mitosis, from 295 ± 11 nm (SD, *n* = 28 cells) to 305 ± 10 nm (SD, *n* = 43 cells) (Fig. 1b), suggesting that myosins are more aligned with the plasma membrane in the mitotic compared with the interphase cortex. As a reference, we also measured the projected lengths of myosins in stress fibers in spread interphase cells, where minifilaments are expected to be parallel to the plasma membrane. The average minifilament projected length was 317 ± 6 nm (SD, *n* = 39 cells) in stress fibers, significantly higher than in the cortex (Fig. 1b). These values are comparable to myosin minifilament lengths measured in electron microscopy images^21^ (~330 nm), suggesting that the stress fiber minifilaments are indeed almost parallel to the plasma membrane. Assuming that minifilament size does not change during the cell cycle, our measurements indicate that the average angle of myosin minifilaments with respect to the plasma membrane decreases from ~20° to ~15° between interphase and mitosis. In the simplest approximation in which every minifilament contributes equally to tension generation, such a change in angle would result in <5% increase in tension. Thus, the mitotic tension increase is not likely to be explained by a change in cortical myosin minifilament orientation alone.

### Myosin motors do not penetrate the entire actin cortex in interphase cells

We then asked how myosin was distributed relative to the cortical actin network. We first imaged cortical actin and myosin with respect to the plasma membrane using confocal microscopy and adapted a subresolution image analysis pipeline we previously developed for assessing actin cortex thickness^22^. Subresolution analysis (see “Methods”) of the confocal images indicated that the cortical myosin layer was thinner than the actin layer in interphase cells (Supplementary Fig. 3a–d). This suggests that myosin does not fully overlap with actin at the cortex, and that in interphase cells, a portion of the actin cortex close to the plasma membrane may be devoid of myosin motors (Supplementary Fig. 3e).

**Fig. 2:**
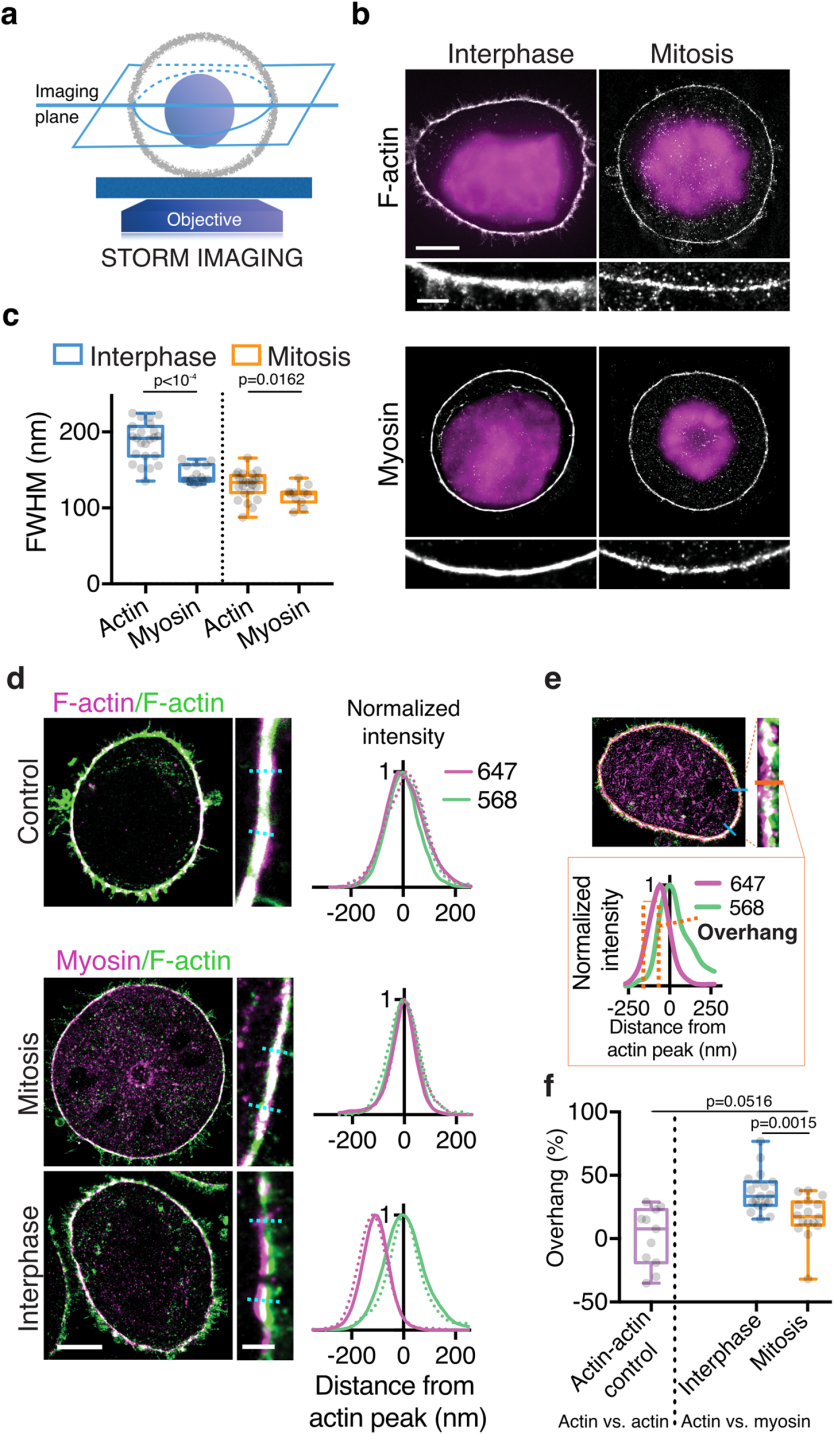
Myosin does not penetrate the entire actin cortex in interphase. **a** Left: Schematic representation of the STORM imaging setup. STORM imaging was performed in the cellular midplane, allowing for high signal-to-noise ratio (SNR) owing to a lack of cell material above and below the equatorial cortex. **b** Representative single-color dSTORM images of cortical F-actin (AlexaFluor 647 Phalloidin) and myosin (overexpressed MRLC-GFP labeled with anti-GFP nanobody-AlexaFluor 647) in rounded interphase and mitotic HeLa cells. Magenta: DAPI staining, used to distinguish the cell cycle phase. **c** Full width at half maximum (FWHM), as an estimate of cortical thickness, of actin and myosin transversal intensity profiles in rounded interphase and mitotic cells (actin: *n* = 23 and 24 cells, 3 and 4 independent experiments, respectively; myosin: *n* = 14 and 11 cells, 4 and 2 independent experiments, respectively). **d** Representative dual-color dSTORM images. Top: Control: F-actin labeled with AlexaFluor 568 Phalloidin (green) and AlexaFluor 647 Phalloidin (magenta) in a rounded interphase cell. Bottom: mitotic and rounded interphase cells expressing MRLC-GFP and labeled with an anti-GFP nanobody–AlexaFluor 647 (myosin, magenta) and AlexaFluor 568 Phalloidin (F-actin, green). Graphs: examples of normalized transversal intensity profiles across the cortex, sampled at blue dotted lines in zoomed-in images. To assess myosin localization at the cortex itself, we focused on regions along the cell contour displaying low concentrations of microvilli (see Methods). **e** Quantification of cytoplasmic overhang in dual-color dSTORM images. The cytoplasmic overhang is the percentage of the FWHM in the 647 channel that does not overlap with the FWHM in the 568 channel. **f** Cytoplasmic overhang for the conditions depicted in **d**. Actin/Actin control: *n* = 11 cells, 4 independent experiments, myosin/actin in rounded interphase and mitotic cells: *n* = 19 and 16 cells, 5 and 3 independent experiments respectively. All boxplots show 25th–75th percentiles, the median, and whiskers from minimum to maximum. Statistics: Mann–Whitney U test (two-tailed).

Confocal microscopy however, does not offer sufficient spatial resolution to directly assess the level of overlap of actin and myosin at the cortex. Therefore, we turned to Single-Molecule Localization Microscopy, which offers a lateral spatial resolution up to 10 times higher than that of confocal microscopy, enabling the relative localization of cortical actin and myosin to be directly examined. First, we imaged cortical actin and myosin separately, at the equatorial cross-section of the cell, using single-color dSTORM (Fig. 2a, b). The thickness of the cortical layers of actin and myosin was estimated by measuring the full width at half maximum (FWHM) of their transversal intensity profiles across the cell boundary, focusing on regions where the cortex was well defined, without excessive microvilli (see Methods). We found that the cortical actin layer was ~30% thinner in mitosis than in interphase (Fig. 2c), consistent with our previous measurements in live HeLa cells using subresolution analysis of confocal images^12^ (Supplementary Fig. 3d). The cortical myosin layer was also thinner in mitosis than in interphase (Fig. 2c). However, whereas in mitosis the actin and myosin FWHMs were comparable, consistent with the two layers overlapping, in interphase, the myosin cortex was significantly thinner than the actin cortex (Fig. 2c), in line with our confocal microscopy-based assessment (Supplementary Fig. 3d). This observation further suggests that cortical myosin does not spread through the entire thickness of the actin cortex in interphase.

We next developed a protocol for two-color dSTORM to image both actin and myosin in the same cell (Fig. 2d, see Methods). We corrected for sample drift and chromatic aberrations (Supplementary Fig. 4), and verified that our analysis pipeline was effective by showing that the cortical intensity profiles of F-actin labeled with two spectrally distinct fluorophores overlapped (Fig. 2d and Supplementary Fig. 5a). We then examined the relative localization of actin and myosin at the cortex. In mitosis, we found no significant shift between the peaks of the cortical distributions of actin and myosin (Supplementary Fig. 5a). In contrast, in interphase cells, cortical myosin was shifted toward the cytoplasm compared with actin (Supplementary Fig. 5a) and a thin actin layer near the plasma membrane was largely devoid of myosin staining; the myosin layer appeared to extend into the cytoplasm beyond the actin cortex (Fig. 2d). To quantify this incomplete penetration, we measured the portion of the myosin FWHM that did not overlap with the actin FWHM (termed overhang, Fig. 2e). Interphase cells displayed an ~35% cortical myosin overhang, whereas no significant overhang was observed in mitotic cells (Fig. 2f). Taken together, these results indicate that myosin-II motors do not completely penetrate the cortical actin layer in interphase cells.

### Incomplete penetration of myosin into the cortex could be due to steric hindrance

**Fig. 3:**
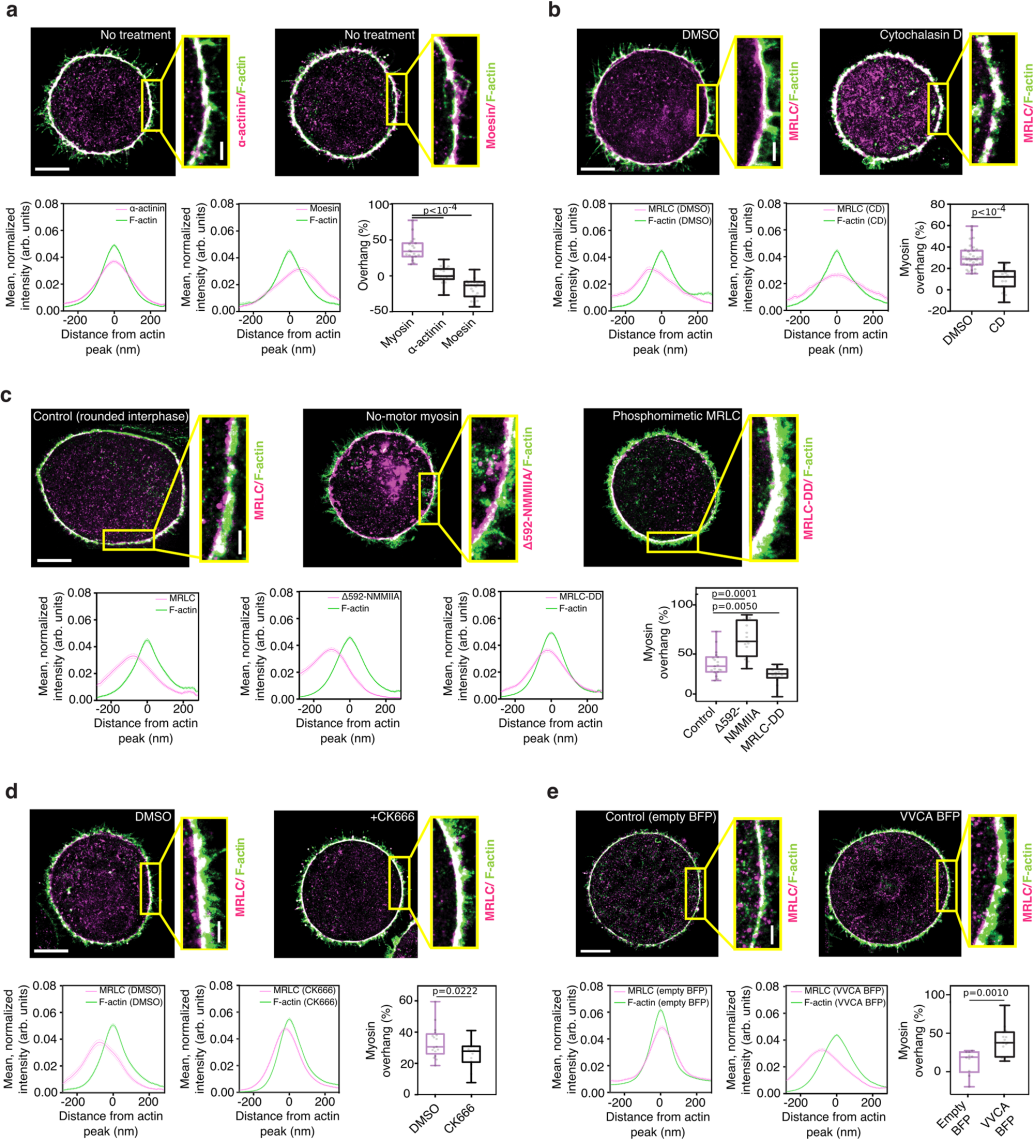
Steric effects limit the penetration of myosin motors into the actin cortex. **a** Top: representative dSTORM images of cortical F-actin (green) and α-actinin or moesin (magenta) in rounded interphase HeLa cells. Bottom: representative cortical intensity profiles of F-actin (green) and α-actinin or moesin (magenta), and cytoplasmic overhang of myosin (data replotted for reference from Fig. 2f), moesin and α-actinin (right-hand side); *n* = 19, 23, and 21 cells; 3, 4, and 4 independent experiments respectively. **b** Top: representative dSTORM images of cortical myosin (magenta) and F-actin (green) in rounded interphase HeLa cells treated with DMSO (control) or 50 nM CD. Bottom: representative cortical intensity profiles of F-actin (green) and MRLC (magenta) and the corresponding cytoplasmic overhang (right-hand side), *n* = 35 and 19 cells; 3 and 3 independent experiments, respectively. **c** Top: representative dSTORM images of cortical F-actin (green) and various myosin mutants (magenta) in HeLa cells expressing MRLC-GFP (control), EGFP-MRLC2-DD, and ΔN592 NMM HCIIA-GFP. Bottom: representative cortical intensity profiles of F-actin (green) and myosin (magenta), and the corresponding cytoplasmic overhang (right-hand side); *n* = 19, 14, and 15 cells; 5, 5, and 4 independent experiments, respectively. Control condition is replotted from Fig. 2f (interphase). **d** Top: representative dSTORM images of cortical F-actin (green) and myosin (magenta) in rounded interphase HeLa cells treated with DMSO and 20 µM CK666. Bottom: representative cortical intensity profiles of F-actin (green) and myosin (magenta) and the corresponding cytoplasmic overhang (right-hand side); *n* = 17 and 14 cells, 3 and 3 independent experiments respectively. **e** Top: Representative dSTORM images of cortical F-actin (green) and myosin (magenta) in mitotic HeLa cells expressing an empty BFP vector (control) and VVCA-BFP. Bottom: representative cortical intensity profiles of F-actin (green) and myosin (magenta) and the corresponding cytoplasmic overhang (right-hand side); *n* = 10 and 15 cells in 2 and 3 independent experiments, respectively. In all panels: the intensity profiles are mean profiles of all analyzed bins along the contour of a representative cell, where the position of the actin peak was set to *x* = 0 nm in each bin; as a result, the intensity profiles in the non actin channel (α-actinin, moesin, and myosins) appear wider than if analyzed individually. All boxplots show 25th–75th percentiles, the median, and whiskers from minimum to maximum. Scale bars, 5 µm and 0.5 µm. Statistics: Mann–Whitney U test (two-tailed) or Welch’s *t*-test (two-tailed).

Myosin minifilaments are ~300 nm long with minifilament heads of ~50 nm in diameter^19,21^, considerably larger than the reported mesh size of the cortical actin network (<50 nm in HeLa cells^12^). We thus hypothesized that steric hindrance could limit myosin penetration into the actin cortex. To test this hypothesis, we first investigated the localization of cortical proteins smaller than myosin II, within the interphase cortex, via dSTORM. We chose the actin crosslinker α-actinin, which is ~35 nm (Ref. ^23^), and the cortex-membrane linker moesin, which is ~14 nm (Ref. ^24^). No shift between the peaks of cortical actin and α-actinin distributions and no cytoplasmic overhang were observed, with the cortical distributions of α-actinin and F-actin essentially overlapping (Fig. 3a and Supplementary Fig. 5b). The peak of the moesin distribution was shifted toward the plasma membrane, compared with the peak of the actin distribution (Supplementary Fig. 5b), and the overhang was negative (Fig. 3a), consistent with moesin binding the plasma membrane in addition to actin. These observations suggest that small proteins, such as α-actinin and moesin, can penetrate the entire actin cortex in interphase cells, in striking contrast with the physically larger myosin-II minifilaments.

If the small size of the actin mesh sterically limits myosin penetration into the cortex, artificially creating holes in the actin meshwork should facilitate myosin penetration. We thus treated interphase cells with Cytochalasin D (CD), an inhibitor of actin polymerization, which at low concentrations induced large holes in the cortex without completely depolymerizing the actin network (Supplementary Fig. 5c). We examined the outer surface of the actin cortex using Scanning Electron Microscopy (SEM), and found that the average pore area of the actin meshwork in interphase cells was indeed increased upon mild CD treatment (Supplementary Fig. 5d). Strikingly, CD treatment increased the overlap between cortical actin and myosin in interphase cells: the actin–myosin peak-to-peak distance was reduced (Supplementary Fig. 5c) and the cytoplasmic myosin overhang significantly decreased (Fig. 3b). Taken together, this suggests that decreasing the density of cortical actin facilitates myosin penetration into the cortex, supporting the steric hindrance hypothesis.

### Myosin activity and actin organization control myosin penetration into the cortical actin network

We then investigated how myosin penetration into the cortex is regulated. We reasoned that if myosin minifilaments are too large to passively diffuse into the cortex, they may enter the cortical network by actively walking on actin filaments. We thus asked whether myosin activity affected actin–myosin overlap at the cortex. We overexpressed NMM HC IIA ΔN592, a truncated NMM HC IIA construct lacking the motor domain, which thus has no motor activity^25^, and a phosphomimetic MRLC mutant, MRLC2-DD, as MRLC phosphorylation promotes myosin activity^18^, and assessed its localization with respect to cortical actin in interphase cells. Both mutants were recruited to the actin cortex, and their expression did not significantly affect cortical actin thickness (Supplementary Fig. 5e). Interestingly, the two mutants displayed different levels of cortical penetration (Fig. 3c). The peak of the cortical distribution of the no-motor myosin mutant was significantly displaced toward the cytoplasm (Supplementary Fig. 5f) and the mutant displayed a ~twofold larger overhang compared with controls (Fig. 3c). Conversely, the phosphomimetic MRLC cortical distribution was shifted towards the plasma membrane (Supplementary Fig. 5f) and displayed a reduced overhang compared with controls (Fig. 3c). Thus, motor activity appears to control the extent of myosin penetration inside the actin cortex.

We next considered if actin network architecture also regulates cortical myosin penetration. For this, we interfered with the activity of the Arp2/3 complex, which controls actin filament branching and has been shown to contribute to actin nucleation at the cortex^26^. We used the Arp2/3 inhibitor CK666^27^ to decrease Arp2/3 activity. Interestingly, Arp2/3 inhibition increased myosin penetration into the interphase cell cortex: both the actin–myosin peak-to-peak distance and the cytoplasmic myosin overhang were reduced compared with controls (Fig. 3d and Supplementary Fig. 5g). Examination of the outer surface of the interphase cortex using SEM showed that CK666 treatment leads to a slight increase in the average actin pore area compared with controls (Supplementary Fig. 5d), consistent with a decrease in cortical density. Taken together, this suggests that decreasing branching and actin density in the cortical actin network could facilitate myosin cortical penetration.

Finally, we investigated the effect of overactivating the Arp 2/3 complex on the localization of myosin within the mitotic cortex by overexpressing a membrane-targeted VVCA, a domain of N-WASP that activates Arp2/3^28^. VVCA overexpression in mitotic cells, where actin and myosin normally overlap at the cortex, strongly increased the actin–myosin peak-to-peak distance and the cytoplasmic myosin overhang (Fig. 3e and Supplementary Fig. 5h). These results indicate that increasing actin branching could limit myosin cortical penetration. Taken together, our observations suggest that myosin penetration into the cortex is likely sterically limited, and is enhanced by increasing myosin activity and by decreasing cortical actin network density.

### Increasing myosin penetration into the actin cortex increases cortical tension

**Fig. 4:**
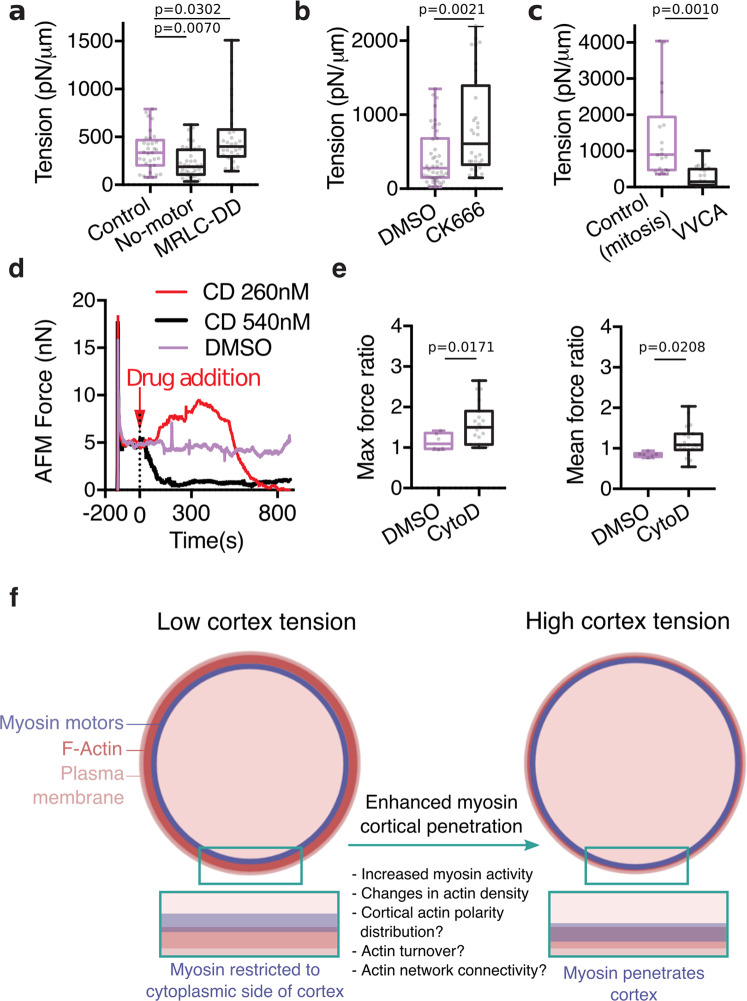
Changes in cortical tension upon changes in myosin penetration into the cortex. **a–c** Cortical tension measurements for **a**, interphase cells expressing myosin mutants (ΔN592 NMM HCIIA (no motor) and MRLC-DD), **b** 20 µM CK666 treated interphase cells, and **c**, mitotic cells expressing VVCA. *n* = 33, 39, and 30 cells in 2, 3, and 2 independent experiments, respectively, in **a**, *n* = 44 and 32 cells in 3 and 2 independent experiments respectively in **b** and *n* = 18 and 22 cells in 2 and 2 independent experiments, respectively, in **c**. **d**, Example force curves exerted by a compressed rounded interphase HeLa cell on an AFM cantilever (AFM force as a readout of cortical tension) upon addition of DMSO (control) and different doses of CD to the sample. After a force peak upon initial cell compression, the AFM force was first left to relax, until a plateau was reached, DMSO or CD were then added (*t* = 0 s on graph). Source Data are provided as a Source Data file. **e** Corresponding quantification of all AFM experiments (performed as described in (**d**)) showing the maximum and mean AFM forces after addition of DMSO (control) and 260 nM CD to the dish, normalized to the AFM force before treatment. *n* = 6 and 19 cells, 2 and 4 independent experiments, respectively. **f** Schematic depicting the possible mechanisms controlling myosin penetration into the actin cortex. All boxplots show 25th–75th percentiles, the median, and whiskers from minimum to maximum. Statistics: Mann–Whitney U test (two-tailed) or Welch’s *t*-test (two-tailed).

Myosin motors that incompletely overlap with actin are unlikely to efficiently generate contractile stresses. We thus asked if changing the actin–myosin overlap at the cortex affected cortical tension. Tension was measured using an Atomic Force Microscopy (AFM) assay^12^. We found that cortical tension was decreased in cells expressing the no-motor myosin mutant and increased in cells expressing phosphomimetic MRLC (Fig. 4a), directly correlating with the levels of cortical myosin penetration (Fig. 3c). However, changes in contractile stress generation upon expression of myosin mutants likely affect tension generation, regardless of changes in actin–myosin overlap. We thus asked how perturbations that modulate myosin penetration without directly interfering with myosins affect tension. We found that Arp2/3 inhibition in interphase cells, which increases myosin penetration into the cortex (Fig. 3d), increased cortical tension (Fig. 4b). In contrast, VVCA expression in mitotic cells, which decreases myosin penetration (Fig. 3e), decreased cortex tension (Fig. 4c). None of the treatments significantly altered the level of MRLC phosphorylation (Supplementary Fig. 6), indicating that changes in myosin activity do not account for the observed changes in tension. Together, these results demonstrate that the level of overlap between actin and myosin at the cortex correlates with cortical tension.

Finally, we asked whether increasing myosin penetration into the cortex acutely would lead to an immediate increase in tension. To this aim, we made use of the observation that treatment with low doses of CD decreases actin cortex density and increases cortical actin–myosin overlap (Fig. 3b and Supplementary Fig. 5c). Accordingly, we monitored cortical tension upon CD addition to a cell maintained under an AFM cantilever (Fig. 4d, e). No tension increase was observed upon addition of DMSO (control), which did not affect tension, or of high doses of CD, which led to an immediate drop in cortical tension likely resulting from quick disruption of the cortical actin network (Fig. 4d). Strikingly, upon addition of lower doses of CD, we observed a transient increase in cortical tension, followed by a drop in tension, presumably due to disruption of the actin cortex upon prolonged exposure to the drug (Fig. 4d, e). Taken together, our observations strongly suggest that increasing the level of penetration of myosin motors into the actin cortex directly increases cortical tension.

## Discussion

We investigated how the spatial arrangement of myosin motors at the cortex affects cortical tension. We first demonstrate that the increase in cortical tension observed between interphase and mitosis cannot be solely ascribed to increased myosin levels or activity (Supplementary Fig. 1). Furthermore, our SIM imaging suggests that while cortical myosin minifilaments slightly reorient toward the plasma membrane in mitosis, the change in filament angle is small and would have a negligible effect on tension (Fig. 1). Of note, our analysis of myosin orientation relies on 2D projections assuming that minifilament length remains constant (Fig. 1b). In the future, 3D SIM imaging should help fully address myosin minifilament orientation in the cortex. However, even if in the mitotic cortex, higher local stresses might lead to myosin minifilament lengthening compared with interphase, as has been proposed in a theoretical model^29^, this would only result in an even smaller change in angle between interphase and mitosis. Thus, it is unlikely that myosin minifilament reorientation significantly contributes to the increase in cortical tension between interphase and mitosis.

Instead, we suggest that modulating the extent of actin–myosin overlap at the cortex is a key mechanism controlling cortical tension (Figs. 3, 4). We show that myosin minifilaments do not fully penetrate into the cortical actin network in interphase cells, which display low cortical tension, while the significantly smaller proteins α-actinin and moesin do. Indeed, we show that in interphase cells, myosin displays a significant overhang, with the most cytoplasmic part of the cortical myosin layer not overlapping with cortical actin. We further show that the overlap between actin and myosin at the cortex can be tuned by modulating myosin activity or cortical actin organization, and that cortical tension correlates with the level of actin–myosin overlap. One possibility is that the myosin overhang reflects that some of the cortical myosin minifilaments are bound to the cortex by only one end with the other end remaining free in the cytoplasm; myosin minifilaments in such configurations would not strongly contribute to cortical tension generation. Altogether, we suggest that myosin entry into the cortex is limited by steric effects, due to the large size of myosin minifilaments compared with the cortical actin mesh size. Interestingly, it has been previously shown that myosin becomes restricted to the cytoplasmic side of the cortex during meiosis I in mouse oocytes, and that this is associated with a strong decrease in cortical tension^30^. In light of our findings, it is likely that steric effects related to the size of myosin minifilaments drive the myosin cortical exclusion in the mouse oocyte. More recently, through a combination of theoretical modeling and super-resolution microscopy, direct evidence of stratification of actin and myosin II in an in vitro system has been shown^31^. Taken together, increasing evidence points to the importance of steric effects in controlling the organization and mechanics of actomyosin networks.

Importantly, while our experimental perturbations of cortical actin organization suggest that increasing the pore size of the cortical actin network facilitates myosin penetration into the cortex (Fig. 3, Supplementary Fig. 5), a mere change in cortex density is unlikely to account for the enhanced actin–myosin overlap we observe in mitotic cells (Fig. 2f). Indeed, we have previously shown that the actin cortex meshsize, as assessed by SEM, does not considerably change between interphase and mitosis^12^. Only the outer surface of the cortex, in direct contact with the plasma membrane, is accessible to SEM imaging. Nonetheless, this suggests that additional mechanisms control the enhanced penetration of myosin motors into the cortex in mitotic cells.

It is indeed likely that other aspects of actin cortex architecture, such as the polarity distribution of actin filaments and the local dynamics of network turnover, affect myosin entry into the cortex (Fig. 4f). Indeed, myosin minifilaments walk toward actin plus (+) ends; the distribution of actin filament + ends across the cortex is thus a key parameter likely to control the overlap of actin and myosin in the cortex. Similarly, the turnover dynamics of actin crosslinkers, which control the network meshsize and could also act as roadblocks limiting myosin minifilament movement, is another key candidate for myosin overhang regulation. Taken together, we expect that the extent of the myosin overhang depends on the interplay between actin nucleators, turnover regulators, capping proteins, crosslinkers, and other regulators of actin polarity and cortical network organization. Tools to investigate the structural organization of cortical actin and its key regulators are still limited, but the rapid progress of super-resolution approaches will make more holistic investigations of how cortex architecture and dynamics control myosin cortical localization, and their resulting tension-generating ability, increasingly accessible.

While our study strongly suggests that enhanced actin–myosin overlap at the cortex contributes to the tension increase associated with mitosis, it also highlights that this enhanced overlap cannot account for the full extent of the tension increase. Indeed, while overexpression of constitutively active MRLC (MRLC-DD) leads to an actin–myosin overlap in interphase comparable to that of control mitotic cells (Fig. 3c), tension is only slightly increased (Fig. 4a). Previous studies have shown that at a given myosin level, changes in actin filament length and the degree of crosslinking also strongly affect cortical tension generation (reviewed in Ref. ^10^). In particular, a thinning of the cortex in mitosis has been shown to be important for the mitotic tension increase^12^. No such cortex thinning is observed upon MRLC-DD overexpression (Supplementary Fig. 5e), suggesting that the presence of constitutively active MRLC does not affect the organization of the cortical actin network. It is also very likely that other features of the cortex could contribute to the regulation of cortical tension upon mitotic entry, including changes in myosin minifilament assembly dynamics, as well as in actin filament polarization and turnover, all of which would be expected to affect contractility generation. Taken together, the mitotic tension increase likely results from changes in cortex composition and nanoscale organization at multiple levels.

More generally, our findings will have strong implications for models of tension generation, which typically assume that myosins are homogeneously distributed throughout the cortical layer. A myosin distribution restricted toward the cytoplasm and only partially overlapping with actin is likely to significantly affect how much tension is generated. Furthermore, a non-uniform actin network architecture across the cell could lead to differential myosin penetration and thus tension gradients. To what extent actomyosin overlap at the cell cortex contributes to the formation of cortical tension gradients driving specific cell shape changes will be an important question for future studies. More generally, our findings highlight that protein size could act as a major structural regulator of architecture and function in cytoskeletal networks, and possibly in other dense cellular structures.

## Methods

### Cell culture, synchronization, and experimental treatments

HeLa TDS cells were a gift from the MPI-CBG Technology Development Studio (TDS, Dresden, Germany). The S-HeLa cell line was derived from HeLa TDS by constant culturing on low-adherent flasks (Corning)^12^. Cells were cultured in DMEM (Gibco, Invitrogen) containing 10% fetal bovine serum (FBS, Life Technologies), 1% penicillin/streptomycin (Life Technologies), and 1% glutamine (Life Technologies) at 37 °C + 5% CO_2_, and were periodically tested in-house for mycoplasma contamination.

HeLa cells were synchronized in interphase by overnight (13–16 h for regular HeLa cells; 16–24 h for confocal imaging experiments with S-HeLa cells) incubation with 2 mM thymidine (Sigma). A cell population enriched in prometaphase cells was obtained by treating cells with 2 μM S-trityl-L-cysteine (STLC, Sigma) overnight (13–16 h) for regular HeLa cells, or 5–7 h for confocal imaging experiments with S-HeLa cells. For observations of the cortex, synchronized cells were rounded by application of Trypsin-EDTA (0.05%, Thermo Fisher Scientific), spun down to remove Trypsin, and resuspended in culture medium. For the confocal measurements of cortical actin and myosin thickness (Supplementary Fig. 3), cells were pretreated for ~30 min with methyl-β-cyclodextrin (MBCD, 5 mg/ml, Sigma-Aldrich) in order to reduce microvilli that could interfere with thickness analysis^22^. For CK666 treatment, rounded cells were incubated for 30 min at 37 °C + 5% CO_2_ in the presence of 20 μM of CK666 (#182515-25MG, Merck Millipore). For Cytochalasin D (CD) treatment, unless indicated otherwise, rounded cells were incubated for 10 min at 37 °C + 5% CO_2_ with 50 nM CD (Sigma).

### Cell transfection, synchronization, and plasmids

HeLa TDS and S-HeLa cells were transfected using lipofection. For HeLa TDS, ~1×10^6^ cells were plated per well (in a 6-well plate, VWR) and transfected after 5–6 h. Cells were transfected with 1.2 μg of DNA using Lipofectamine 2000 (Life Technologies) following the manufacturer’s instructions. After 24 h, cells were synchronized as described above for further experiments.

For S-HeLa cells, ~500,000 cells were plated in 0.5 ml of DMEM in low-attachment 24-well plates (Corning) ~16 h before transfection. On the next day, cells were spun down and the medium was exchanged for antibiotic-free DMEM. Plasmid DNA and Lipofectamine 2000 (Life Technologies) were each diluted in 50 μl of Opti-MEM (Life Technologies) separately, and then mixed after 5 min, incubated for ~45 min at room temperature, and then added to the cells. About, 0.3–0.7 µg DNA plasmid was used for lipofection. The transfected cells were imaged the next day (after 16–24 h).

The myosin constructs EGFP-NMM HC IIB and NMM HC IIB-N18-tdTom, used for SIM imaging of myosin minifilaments, were kind gifts from John Hammer (NIH, Bethesda) and Jordan Beach (Loyola University)^20^. The myosin-regulatory light chain construct EGFP-N1-MRLC2 and the phosphomimetic EGFP-N1-MRLC2^T18DS19D^ (EGFP-MRLC2-DD) were a kind gift from the Hosoya lab^32^. The truncated ΔN592 NMM HC IIA-GFP construct was generated by PCRing the C-terminal portion of NMM HC IIA downstream of the first 592 amino acids flanked by restriction sites, and inserting this fragment into a pEGFP-C1 (Clonetech) vector using restriction digests. Membrane-targeted EBFP2 (control construct) was generated from palmytoylated ECFP-C1 (ECFP-MEM-C1, Clontech) by swapping ECFP for EBFP2 by restriction digests and ligation. The VVCA domain was PCRed from N-WASP with flanking restriction sites and inserted into the MCS in-frame with EBFP2 and the palmytoylation signal. EGFP-CAAX was a gift from John Carroll; BFP-CAAX was obtained by replacing EGFP with BFP in EGFP-CAAX. The GFP-actin construct was obtained as described in Ref. ^12^.

### Immunostaining for confocal imaging

Fixation and staining were carried out in glass-bottom dishes (#HBST-3522, Willco Wells). Resuspended cells were centrifuged for 4 min at 133 × *g* onto poly-l-lysine- (P4707-50ML, Sigma) coated coverslips of the glass-bottom dish. Cells were fixed and permeabilized simultaneously in 4% methanol-free formaldehyde (FA, Fisher Scientific, prewarmed at 37 °C), 0.5% Triton in Cytoskeleton Buffer with Sucrose (CBS, 10 mM MES, 138 mM KCl, 3 mM MgCl_2_, 2 mM EGTA, and 4.5% sucrose w/v, pH 7.4) for 20 min at room temperature. The amount of sucrose in the CBS was adjusted to make it iso-osmolar to DMEM and thus avoid changing cell volume because of osmotic shock at fixation. Fixed cells were next washed three times with 0.05% Triton in PBS and blocked for 20 min in blocking solution (1% BSA, 10% goat serum in PBS). Blocked cells were immunostained with primary antibodies: α-phospho-MRLC2 (Ser19) (rabbit, #3671 S, Cell Signaling Technology); α-phospho-MRLC2 (Thr18/Ser19) (rabbit, #3674, Cell Signaling Technology); α-MRLC (mouse, M4401, Sigma); α-MRLC (rabbit, #3672, Cell Signaling Technology); and α-MYH9 (rabbit, #14971001, Covance) or α-MYH10 (rabbit, #14942501, Covance), overnight at 4 °C at a ratio of 1:100 v/v in blocking solution. Cells were then washed five times with 0.05% Triton in PBS and incubated in blocking solution for 10 min before staining with secondary antibodies α-mouse Alexa Fluor 488 (A-21202, Invitrogen, 1:200) or α-rabbit Alexa Fluor 488 (A-21206, Invitrogen, 1:200), for 1 h in blocking solution. For staining F-actin and DNA, phalloidin Alexa Fluor 568 (1:200, Life Technologies) and DAPI (1:1000), respectively, were added during the secondary antibody staining. Stained cells were subsequently washed five times with 0.05% Triton in PBS before being embedded in the mounting medium Vectashield (Vector Laboratories), sandwiched by adding another glass coverslip on top, and sealed with nail polish.

### Confocal imaging and image analysis for cortical myosin levels

Cells fixed and stained as described above were imaged using a confocal microscope (Leica TCS SP8 STED 3X, objective x40 oil, NA 1.3) with a high-sensitivity detector (HyD) equipped with the standard Leica LAS X software. For each acquisition, actin, myosin, and DAPI were imaged. Images of cortex components were first corrected for background signal. Images were approximated as the sum of a nonhomogeneous background, and of the fluorescence of specific and unspecific antibodies binding to the cortex. The nonhomogeneous background signal is the combined result of a constant offset of the camera for each pixel value, and of frame-specific features, including out-of-focus cells, cellular debris, and autofluorescence of the mounting medium. We estimated and subtracted the background using a rolling-ball algorithm using the function *imtophat* in MATLAB. Briefly, a sphere with a large radius compared with the thickness of the cortex is “rolled over” the intensity map of the raw image and the bottom surface of the sphere at every point is used to estimate the background at this point. The sphere radius was chosen to be 50 pixels, sufficiently high to detect the background without detecting the cortex (which is typically ~8 pixels in width). The background-subtracted image of the actin cortex was then segmented using a script custom-written in MATLAB. Briefly, for each image, we ran two independent methods of segmentation sequentially (detailed below). The segmentations belonging to each detected cell were grouped together based on their relative centroid distances. For each group of segmentation candidates, the segmentation that reported the highest total signal intensity along the segmented cell contour was chosen as the optimal segmentation for that given cell.

*Segmentation methods*: For the first segmentation method, we chose a region devoid of cells to estimate a thresholding level as a sum of the mean and the standard deviation of pixel values within that specific region. Using this threshold level, binary masks of cells were then extracted from the original image. For the second segmentation method, cell edges were detected using a Sobel filter and the built-in function *edge* in MATLAB. The resultant edges were filled in to exclude holes and cleaned for noisy signals with built-in MATLAB image processing functions. Segmented cells were next separated via a watershed algorithm, and the cell masks were cleaned again with morphological image processing. Cortex outer and inner edges were detected by identifying the boundaries of dilated and eroded binary masks (using structure element of 5 and 3 pixels, respectively). Finally, the extracted actin cortex outline was used as a mask to measure the intensity of cortical myosin (within a thickness of 8 pixels centered at the cortex outline). The DAPI staining was used to classify cells as interphase or mitotic.

For each independent experiment, an additional control sample was imaged to measure the average cortical signal arising from unspecific binding of secondary antibodies. For this, control cells were prepared in the same fashion of the cells of interest; however, the secondary antibody was added without a primary myosin antibody (Supplementary Fig. 1c). The measured signal of this control sample was then subtracted from the myosin cortical intensities to correct for unspecific secondary antibody staining.

### Imaging and image analysis for confocal analysis of relative actin and myosin localization

Images of S-HeLa cells expressing BFP-CAAX, GFP-Actin, and MRLC2-WT-mCherry were acquired using an Olympus FV1200 confocal microscope with a 63X color-corrected objective (OSC2 PlanApoN, 1.4 N.A.). Image stacks were acquired for each cell around the middle plane (~30–70 slices) with a step size of 0.1 μm. Confocal stacks were corrected for chromatic aberration and processed as in Ref. ^12^. The thickness of the actin cortex, *h*_actin_, was then extracted as in Refs. ^12,22^. Like for actin (see Ref. ^22^ for details), extracting the thickness of the myosin cortical layer from confocal images requires assumptions on the distribution of myosin with respect to the plasma membrane. Assuming that the cortical myosin distribution can be described as a step-like function with the cytoplasmic edge of the distribution aligning with the cytoplasmic edge of the actin cortex (Supplementary Fig. 3a), key features of the myosin linescan (intra- and extracellular background levels, peak intensity value, and position) were fit to a convolution of a sum of step functions. This yielded the thickness of the myosin region, *h*_myo_, which with our set of assumptions, describes the penetration depth of myosin into the cortex. The gap between the membrane position and outer myosin edge (the “myosin-free cortex length” in Supplementary Fig. 3) is then simply the difference between the actin cortex thickness and penetration depth.

### SIM sample preparation and imaging

For live imaging of myosin minifilaments, a soft agar slab (1% of low-melting-point agar (Sigma) in 5% HBSS (Sigma), 40% RPMI (Thermofisher), and 10% FBS (Life Technologies)) was prepared before each experiment and preincubated for ~30 min at 37 °C + 5% CO_2_. The glass-bottom dishes (#HBST-3522, Willco Wells) used for imaging were passivated with 2 mg/ml Pluronic F127 (Sigma) diluted in PBS for 1 h. 10–20 μl of a suspension of rounded cells in cell culture medium (at ~10^6^ cells per ml) were then deposited in each passivated imaging dish, and confined by placing the soft agar slab on top of the suspension drop. The bottom surface of the confined cells was imaged using the SIM mode of a Zeiss Elyra microscope (objective x100, oil, NA 1.46). Multicolor fiducial beads (Tetraspecks, #T7279, Invitrogen) immobilized on a glass coverslip were separately imaged for chromatic shift estimation for each independent experiment.

For imaging of the actin cortex via SIM, suspended cells pretreated with CD or equivalent volume of DMSO (control) were centrifuged for 4 min at 133 × *g* on poly-l-lysine-coated coverslips, fixed, and stained for F-actin with phalloidin preconjugated to Alexa Fluor 568. A z-stack of 0.5-µm depth, with a 126 nm Δz step, was acquired around the contact area between the cell and the glass coverslip. The SIM images were obtained in Fiji, by maximum projection of the z-stack after reconstruction using the Zeiss Elyra ZEN software (Zen black).

### Myosin-minifilament detection and measurement

Two-color SIM images of myosin minifilaments with heads and tails labeled with eGFP and tdTom, respectively, were reconstructed using the Zeiss Elyra ZEN software. Chromatic aberration was estimated from images of multicolor fiducial beads (Tetraspecks, #T7279, Invitrogen) and corrected for using affine transformation in the ZEN software. Reconstructed SIM images were postprocessed with custom-written MATLAB script to identify and measure myosin minifilaments (Supplementary Fig. 2). First, background inhomogeneity, estimated by convolving the SIM image with a Gaussian filter of a large radius (500 pixels, ~10 µm), was subtracted from the SIM image. The background-homogenized image was next deconvolved using the *Parallel iterative deconvolution* plugin to Fiji. The image was next thresholded (with the threshold value set to three times the standard deviation of the intensity of a background area in the image without a cell present) to eliminate low intensity homogenized background. Isolated pixels resulting from white noise were removed by an image-erosion operator. Candidate myosin spots were then identified and subsequently localized with a local peak-detection algorithm. Briefly, a pixel was detected as a peak if its intensity was the local maximum of all its eight direct neighboring pixels. The position of candidate myosin spots was then estimated as the centroid of a 5-pixel-wide square region of interest (ROI) around each detected peak. Myosin minifilament candidates were identified as all pairs of ROI centroids in the green channel separated by 200–400 nm. A minifilament candidate was validated if a red peak could be found within a radius of 50 nm from the candidate minifilament center. Note that analysis was restricted to the central regions of the SIM images, that is, away from the cell periphery where the cell rounds up away from the coverslip and the signal becomes blurred as a result.

### STORM sample preparation and imaging

For STORM imaging, cells transfected with the protein of interest tagged with GFP were first fixed and permeabilized for 6 min in 4% FA, 0.2% Triton in CBS (pH 6.9), and then further fixed for 14 min in 4% FA in CBS. Cells were next washed three times with PBST (PBS supplemented with 0.1% Tween), permeabilized a second time in PBS supplemented with 0.5% Triton for 5 min, washed three times with PBST, and blocked for 10 min in PBST supplemented with 4% BSA. The cells were then stained for 1 h at room temperature with Alexa Fluor 647 anti-GFP nanobody (gb2AF647, Chromotek, 1:600), Alexa Fluor 568 phalloidin (#A12380, Thermo Fisher, 1:200), and DAPI (1:1000) diluted in PBST; of note, the anti-GFP nanobody also labels BFP (for VVCA experiments) and is <3 nm in size. Next, cells were washed five times with PBST and incubated in PBS supplemented with ~0.5–0.7*10^9^ multicolor fiducial beads (100 nm diameter, #T7279, Invitrogen) for 30 min. The cells were then washed gently three times in PBS to remove unbounded fluorescent beads and fixed a second time in 4% FA (in PBS) for 15 min to stabilize fiducial beads that bound to the cell surface. The fiducial beads were used for drift correction (see below). Finally, the cells were washed gently five times in PBS to remove traces of fixatives.

For one-color STORM experiments, the stained cells were embedded in 50% Vectashield (Vector Laboratories), and 50% Tris-glycerol (50 mM Tris pH 7.5, in 95% glycerol). For two-color STORM experiments, immediately before imaging, the dish was supplemented with STORM buffer (50 mM Tris, pH 7.5, 10 mM NaCl, 27 mM MEA, 10% glucose, 34 μg/ml catalase, and 0.56 mg/ml glucose oxidase). A second rounded glass coverslip was then added on top of the medium to form a closed chamber and the samples were sealed with nail polish.

STORM images were acquired using a Zeiss Elyra PS.1 super-resolution microscope (objective x100 oil, NA 1.46) operated in STORM mode at 50-Hz acquisition rate. For two-color STORM, imaging was performed sequentially with the 647 channel acquired first and the 568 channel subsequently; 20,000 frames were acquired at 20–30 ms exposure time, using 300 EMGain and the multiband MBS—561/642 filter set. A coverslip of fiducial multicolor beads was also imaged at the end of each acquisition day, for calculation and correction of chromatic aberration.

### STORM image detection and rendering, lateral drift correction, and chromatic aberration correction

Coordinates and localization uncertainties of single molecules in raw STORM images were determined using the open-source software ThunderSTORM^33^. Briefly, the raw image was filtered with wavelet B-Spline to denoise and enhance the point-source features. The molecules’ positions were approximated as local-intensity maxima within 8-connected neighbors. Molecules with low fluorescent intensities often result from short noise or out-of-focus molecules, and were thus eliminated using a threshold of 1.2 times the standard deviation of the first wavelet level that contains most of the background noise. This threshold was chosen as a compromise for low false detection and high density of the final image, judged by visual inspection. The subpixel coordinates of molecules were then extracted by fitting a ROI of 3 × 3 pixels around each local maximum with a 2D Gaussian, using a weighted least-squares method. The localization uncertainty was estimated per the method of Ovesný^33^. Note that the average localization uncertainty of our STORM images was 20 ± 10 nm (Supplementary Fig. 4a).

Drift, chromatic aberration correction, and super-resolution rendering were performed using a custom-written MATLAB (MathWorks) script, as follows. Drift correction: For two-color STORM, mechanical drift during acquisition was corrected by tracking fiducial beads attached to the cell surface (Supplementary Fig. 4b, c). For one-color STORM images, drift was corrected with a redundant cross-correlation algorithm^34^ using a custom-written MATLAB script as follows. First, we rendered a conventional image from coordinates of localized molecules, without drift correction. Next, we semiautomatically segmented the cell cortex using this draft STORM image and a custom graphical interface (MATLAB). Only the cortical signal was used for cross-correlation calculation. The STORM image dataset was then divided into consecutive time windows of 2000 frames. The coordinates of detected cortical single molecules in each time window were rendered into one image using a 2D histogram with sub-pixels of 20 nm, to represent the position of labeled objects in that time window. Drift between time windows was calculated by cross-correlating images of successive windows. The lateral drift at each time point was then interpolated using a spline method, and was subtracted from coordinates of detected molecules at that given time point.

For two-color STORM datasets, chromatic aberration was corrected based on images of multicolor fiducial beads immobilized on a coverslip (Supplementary Fig. 4d). Bead positions were detected in the 568 and 647 channels and an affine transformation was used to fit the chromatic shift measured, taking into account differences in scaling, translation, and rotation.

The final STORM images were rendered from drift and (when relevant) color-corrected coordinates of all localized molecules as a density map by superimposing all normalized Gaussian distributions with a standard deviation of the localization uncertainty, centered at the molecules’ positions. To avoid artifacts induced by digitizing the image (when decimal values are rounded up to the closest integer), the density map was multiplied with a constant of 100. This constant did not affect the results of any measurements reported.

### Cortex FWHM and cytoplasmic overhang measurements

The cell cortex was detected semiautomatically from rendered STORM images of F-actin. First, we manually selected segments where the cortex was well defined, i.e., in regions that did not display many microvilli or aggregates of fiducial beads, using a custom GUI written in MATLAB. The cortex was then automatically detected by finding the maximum peak of transverse profiles at every pixel along the manual segmentation regions. The refined transverse profiles of the cell cortex were sampled along the automatically detected cortex ROI by drawing sampling lines perpendicular to the tangential line at each point of the selected cortex segments. To this aim, a cubic spline was fitted to the detected cortex and a tangential line was determined at each point. The tangential lines were then used to straighten the cortex, so that transversal intensity profiles could be extracted easily.

The straightened cortices were analyzed in bins of 20 pixels (200 nm) along the cortex. In each bin, the fluorescence peak of the cortical profile was detected and the FWHM of the profile was measured. We verified that cell curvature in the z direction did not significantly affect FWHM measurements; assuming a thickness of the imaging plane of ~200 nm, even for a small cell (5 µm radius), out-of-plane fluorophores would increase the measured FWHM by 2 nm at most.

For assessing the overlap of actin with myosin or other cortical proteins, the distance between the peaks of the intensity profiles of the two proteins of interest was measured (peak-to-peak distance). The cytoplasmic overhang was then defined as the percentage of the FWHM of the cortical profile (of the protein of interest) that falls outside the FWHM of the actin profile (Fig. 2d).

### SEM of the interphase cortex

Cells were synchronized in interphase as described previously (Methods; Cell transfection, synchronization, and plasmids). Prior to collection, synchronized cells were washed x1 in PBS followed by x3 washes in culture medium. A visual confirmation that only spread, interphase cells remained on the culture plates was performed prior to collection. Synchronized cells were then rounded by application of Trypsin-EDTA (0.05%, Thermo Fisher Scientific), spun down to remove Trypsin, and resuspended in culture medium. Resuspended cells were centrifuged for 4 min at 133 × *g* onto poly-l-lysine- (P4707-50ML, Sigma) coated coverslips (Thermo Scientific™ Nunc™ Thermanox™ Coverslips, 174950). For drug conditions, cells were incubated with either 50 nM CD or 20 μM CK666 for 30 mins at 37 °C. Samples were then processed for SEM using a protocol detailed in Ref. ^26^, with slight modifications detailed in Ref. ^12^. Briefly, cells were washed in a cytoskeleton buffer (50 mM imidazole, 50 mM KCl, 0.5 mM MgCl2, 0.1 mM EDTA, and 1 mM EGTA, pH 6.8) prior to fixation and extraction in cytoskeleton buffer containing 0.5% Triton-X and 0.25% glutaraldehyde for 5 min at 37 °C. The samples were then further extracted with 2% Triton-X and 1% CHAPS in cytoskeleton buffer for 5 min at room temperature, followed by three washes in cytoskeleton buffer. Cells were then fixed for 20 min in a solution of 0.1 M sodium cacodylate triphosphate and 2% glutaraldehyde. Samples were then washed three times in deionized water. Fixed and extracted samples were then subject to a 0.1% tannic acid (TA) stain for 5 min and washed four times for 5 mins with deionized water, followed by one wash under agitation for 10 min. Samples were then incubated with 0.1% uranyl acetate for 10 min, followed by four washes of 5 min with deionized water and a final wash under agitation for 10 min. Cells were then dehydrated with serial ethanol dilutions (10–100% in 10% increments for 5 min each) and subsequently dried in a critical point dryer. Samples were coated with iridium and imaged using a FEI Verios 460 scanning electron microscope.

### Western blots

Cells synchronized in interphase were trypsinized and collected. Cells enriched in prometaphase (rounded and loosely adhered to the substrate) were selectively collected by mechanical agitation. Cells were first lysed in 1x sample buffer (312.5 mM Tris pH 6.8, 50% glycerol, 5% SDS, 5% β-mercaptoethanol, and 0.05% bromophenol blue). Lysates were sonicated and incubated at 95 °C for 5 min. Lysates were subjected to SDS-PAGE using 14% Novex Tris-Glycine gels (ThermoFisher). Proteins were then transferred to a 0.2-µm nitrocellulose membrane. The membrane was blocked with Odyssey Blocking Buffer (Licor) for 1 h at room temperature. Primary antibodies used were α-MRLC (1:1000, Cell Signaling #3672), α-phospho-MRLC (Ser 19) (1:500, Cell Signaling #3675), and α-GAPDH (1:5000, 1D4 Novus Biologicals NB300-221). The membrane was incubated with primary antibodies at 4 °C overnight in TBST buffer (TBS + 0.1% Tween) for α-MRLC and α-phospho-MRLC and TBST + 5% milk for α-GAPDH. Membranes were then washed three times for 5 min in TBST. Secondary antibodies used were goat α-mouse Alexa Fluor 680 and goat α-rabbit Alexa Fluor 790 (1:20,000, ThermoFisher A-21058 and A11369, respectively). Incubation with secondary antibodies was performed for 30 min at room temperature in TBST + 5% milk. Membranes were then washed three times for 5 min in TBST and imaged using a Licor Odyssey and the resulting images were analyzed using Image Studio Lite software (Licor). The intensity of each band was measured and proteins of interest were normalized to GAPDH bands in corresponding lanes. Each sample was performed in duplicate. Western blot quantifications are presented as averages of three independent experiments.

### AFM experiments

Synchronized interphase cells were trypsinized, suspended into CO_2_-independent medium (Leibovitz’s medium, ThermoFisher), and plated on glass-bottom dishes (coated with poly-L-lysine for 5 min before the experiment) prior to force measurement. For mitotic cells, cells were cultured in glass-bottom dishes (FluoroDish, World Precision Instruments) for 24 h before being synchronized overnight with STLC, and mitotic cells were recognized based on their rounded shape. For CK666 treatment, the drug was added to the cell suspension and the sample was incubated at 37 °C for 20 min before cell plating. Only cells that were still rounded and had not substantially spread were measured.

Cortical tension measurements were performed using a JPK CellHesion module (JPK Instruments) on an IX81 inverted confocal microscope (Olympus), using tipless silicon cantilevers (ARROW-TL1Au-50) with a nominal spring constant of 0.03 N/m. At the start of each day of experiment, the cantilever sensitivity was calibrated by acquiring a force curve on a glass coverslip, and the spring constant was calibrated by the thermal noise fluctuation method using the JPK software. Constant-height mode was selected and a Z-length parameter of 30 μm was used with a setpoint force of 25 nN. The measurement was carried out by first lowering the tipless cantilever in an empty area next to the cell, in order to detect the position of the substrate. The cantilever was then positioned above a rounded cell of interest, and a compression was applied for 80–120 s, while the force exerted on the cantilever was recorded. After initial force relaxation of 20–30 s, the AFM force attained a plateau and that value was used to extract surface tension. For each cell under AFM compression, one confocal image (Olympus, objective x60 oil, NA 1.42) of the cell membrane labeled with CellMask (#C10046, Thermo Fisher Scientific) at the cell equator was taken, to estimate the maximum cross-sectional area of the cell (see below).

The calculation of cortex tension is based on a previously published method^35^. Briefly, neglecting the ~8° angle of the cantilever with respect to the glass-bottom dish and assuming negligible adhesion between the cell, dish and cantilever, the force balance at the contact point reads

1 $$T=\frac{F\left(\frac{{r}_{{{\rm{mid}}}}^{2}}{{r}_{c}^{2}}-1\right)}{2\pi {r}_{{{\rm{mid}}}}}$$

where *r*_mid_ is the radius of the maximum cross-sectional area of the selected cell, *r*_*c*_ is the radius of the contact area of the cell with the cantilever, and F is the force exerted by the cell on the cantilever. To avoid errors due to direct measurement of *r*_*c*_, the contact radius was calculated using the following formula:

2 $${A}_{c}=2\pi {r}_{c}={A}_{{{\rm{mid}}}}-\left(\frac{\pi }{4}\right){h}_{{{\rm{cell}}}}^{2}$$

where *A*_*c*_ is the contact area between the cell and the cantilever, *A*_mid_ is the cell maximum cross-sectional area, and *h*_cell_ is the cell height. The radius *r*_mid_ was measured manually from a confocal image of the cell equator using Fiji software. The height *h*_cell_ was measured as the difference in z positions of the AFM cantilever when compressing the cell during the force measurement and when in contact with the glass coverslip prior to cell compression.

For the measurement of the transient effect of CD treatments on tension, interphase HeLa cells were trypsinized and resuspended into CO_2_-independent medium (Leibovitz’s medium, ThermoFisher) in 50 mm glass-bottom dishes (#FD5040-100, FluoroDish, World Precision Instruments). Cells were incubated for 20–30 min at 37 °C, to allow cells to weakly adhere to the glass. The set-point force was set at 20 nN and constant-height mode was selected. The cantilever was positioned above the target cell and compression was applied for 1000 s. Once the resulting force reached a constant value (~100 s), 100 µl of Leibovitz’s medium with 260 nM CD, 540 nM CD, or equivalent concentration of DMSO for controls, were slowly added through a Microloader (Eppendorf), at the side of the dish. The initial CD concentrations chosen were higher than those for the experiments depicted in Fig. 3b, as the drug is likely to be significantly diluted by the time it reaches the cell in this setup. The resulting force curves were smoothed with a Savitzky–Golay filter in Python. Finally, we calculated the mean force in 20 s prior to drug addition, and compared this with the mean or the maximum force displayed in 300 s post drug addition.

### Statistical analysis and reproducibility

For each experiment, data distributions were tested for normality using a D’Agostino–Pearson test. Comparisons of means between different conditions were tested with a Welch’s *t*-test (two-tailed, no assumption of variance) if the data passed the normality test, or with the nonparametric Mann–Whitney U test otherwise. *P*-value summaries: *P < 0.05, **P < 0.01, and ***P < 0.001. All statistical tests were performed in Prism (GraphPad Software, Inc).

### Reporting summary

Further information on research design is available in the Nature Research Reporting Summary linked to this article.

## Supplementary information

Supplementary Information

Reporting Summary

## Data Availability

The raw data generated in this study underlying Figs. 1b and 4d are provided in the Source Data file. Images are available from the authors upon request. All further data are available in the article or Supplementary Information. Source data are provided with this paper.

## Source data

Source Data

## Code availability

The software developed for image processing and analysis is available at https://github.com/PaluchLabUCL.
