# Supplementary material for: Extent of myosin penetration within the actin cortex regulates cell surface mechanics
Source: Nat Commun. 2021 Nov 11;12:6511. doi: 10.1038/s41467-021-26611-2 (PMC8586027; doi:10.1038/s41467-021-26611-2)
Supplement: Supplementary file 1 — Supplementary Information [file 41467_2021_26611_MOESM1_ESM.pdf]

Supplementary Information for manuscript entitled

"Extent of myosin penetration within the actin cortex regulates cell surface mechanics"

Authors: Binh An Truong Quang*, Ruby Peters*, Davide A.D. Cassani, Priyamvada Chugh, Andrew G. Clark, Meghan Agnew, Guillaume Charras, Ewa K. Paluch**

* These authors contributed equally
**Correspondence to: ekp25@cam.ac.uk

This PDF file includes:

Supplementary Figures 1-7

Supplementary Fig. 1

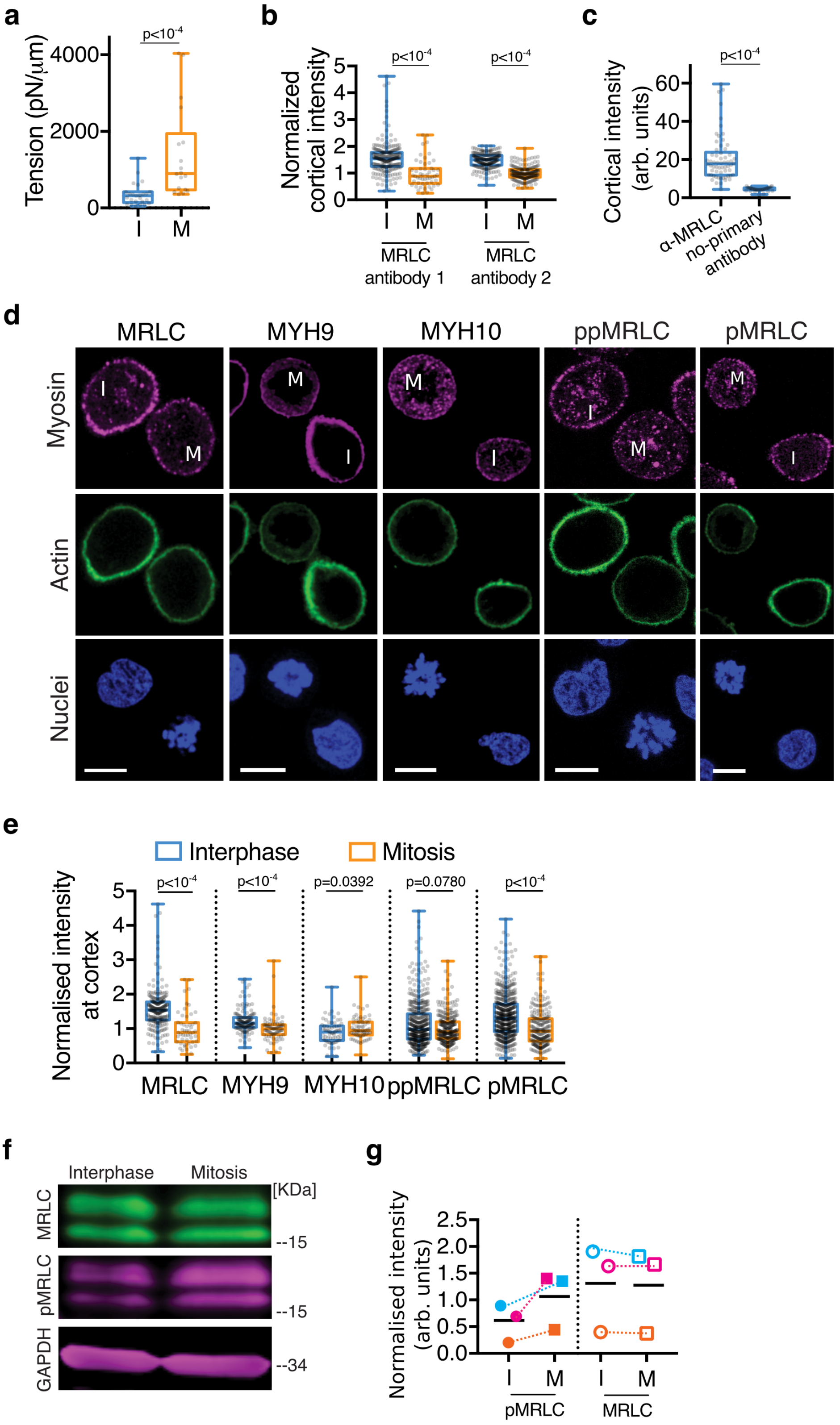

Supplementary Fig. 1: Cortical tension and cortical myosin levels in interphase and mitosis. **a,** Cortical tension in rounded interphase (denoted I throughout) and mitotic (denoted M throughout) HeLa cells, n = 25 and 18 cells, respectively; 2 independent experiments.

Mitotic condition replotted from **Figure 4c**. **b,** Normalized mean intensity of cortical MRLC in rounded interphase and mitotic cells stained with two different MRLC antibodies, n = 293 and 421 cells, respectively; at least 2 independent experiments. **c,** Example control experiment for non-specific binding: cortical intensities in cells stained with an α-MRLC antibody, and in cells subjected to the same staining protocol but without primary antibody staining, n=62 and 34 cells respectively; at least 2 independent experiments. **d,** Representative confocal images of the equatorial plane of rounded interphase and mitotic HeLa cells stained for actin (green), DAPI (blue), and myosin (magenta) probed with different antibodies: α-MRLC, α-MYH9 (HC IIA), α-MYH10 (HC IIB), α-ppMRLC, α-pMRLC. Scale bars, 10 μm. **e,** Quantification of cortical myosin from confocal images exemplified in **d**. To account for variation between different days of experiment, cortical intensities were normalized to the mean of cortical intensities of all mitotic cells measured on a given day; n = 70 to 500 cells per cell stage and condition, at least 3 independent experiments per condition. **f,** Representative fluorescent immunoblot of MRLC (green), pMRLC (Ser19; magenta) and loading control GAPDH (magenta) in interphase and mitosis. Uncropped blots are shown in Fig. S7. **g,** Normalized intensities of pMRLC and MRLC in interphase (circles) and mitosis (squares) exemplified in **d**. All Boxplots show 25th to 75th percentiles, the median, and whiskers from minimum to maximum. Statistics: Mann-Whitney test (two-tailed).

Supplementary Fig. 2

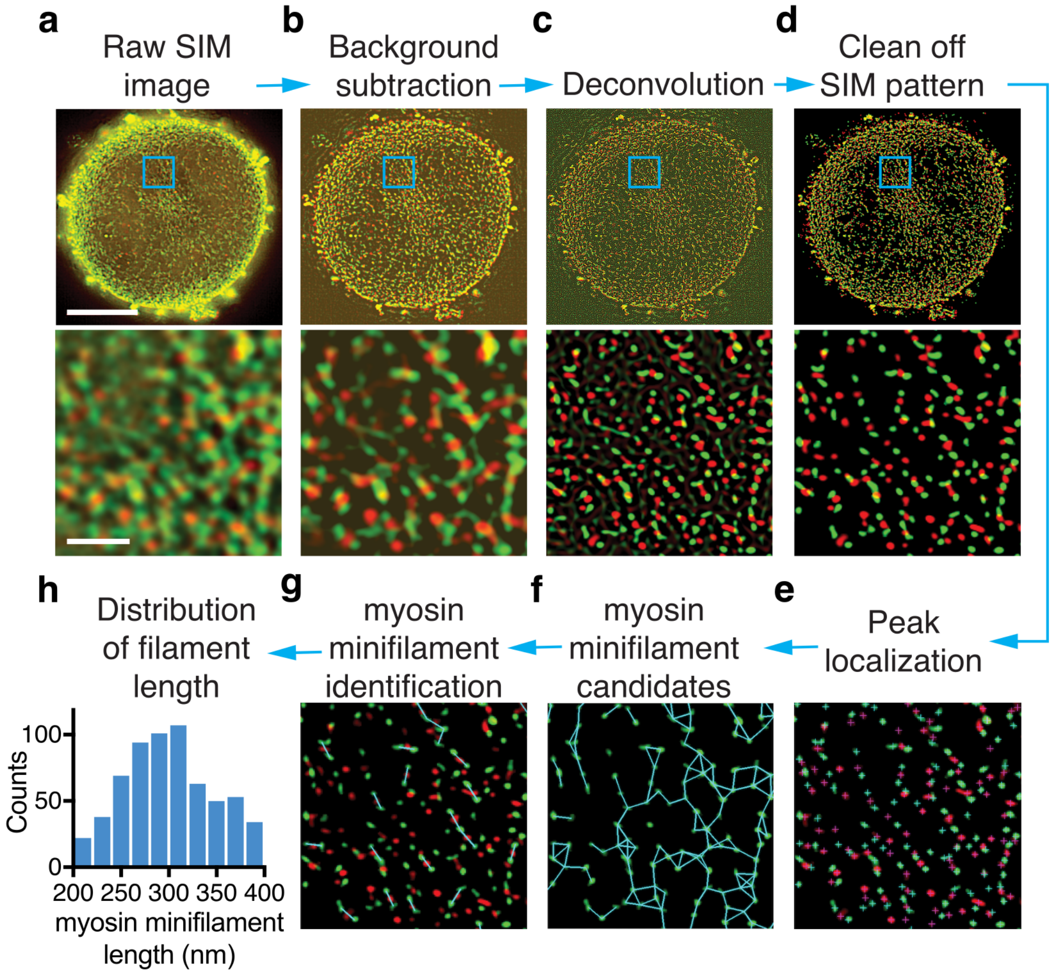

Supplementary Fig. 2: Automatic detection of myosin minifilaments in SIM images of the cortex. a, Exemplar raw SIM image of cortical myosin with heads and tails differentially labeled with eGFP and tdTom respectively, in a mitotic HeLa cell. The cell is compressed under an agar pad such that a large flat cortical surface can be imaged (see Methods). The raw image contains inhomogeneous background signal and SIM reconstruction artifacts that must be corrected for to construct the SIM image for further analysis. **b-h,** Outline of the image processing and myosin minifilament identification analysis pipeline; see Methods for details. First, out-of-focus background in SIM images was subtracted **b**, and images were subsequently deconvolved **c,** using an experimental PSF estimated by imaging a fluorescent bead sample. The artifacts due to SIM image reconstruction and deconvolution were then removed by thresholding **d.** The myosin heads were identified by peak localization **e**, and any two peaks localized in the green channel separated by a distance of 300 ± 100 nm were grouped into a minifilament candidate **f**. The minifilament candidates for which an additional peak in the red channel could be found within a radius of 50 nm from the center of the minifilament candidate were then selected as detected minifilaments, **g**. **h,** Example distribution of detected myosin minifilament length at the surface of the cell presented in **a-d** (defined as the Euclidean distance between the two identified green peaks). Scale bars 10 µm (topmost images **a-d**) and 0.5µm (bottommost images **a-d**).

Supplementary Fig. 3

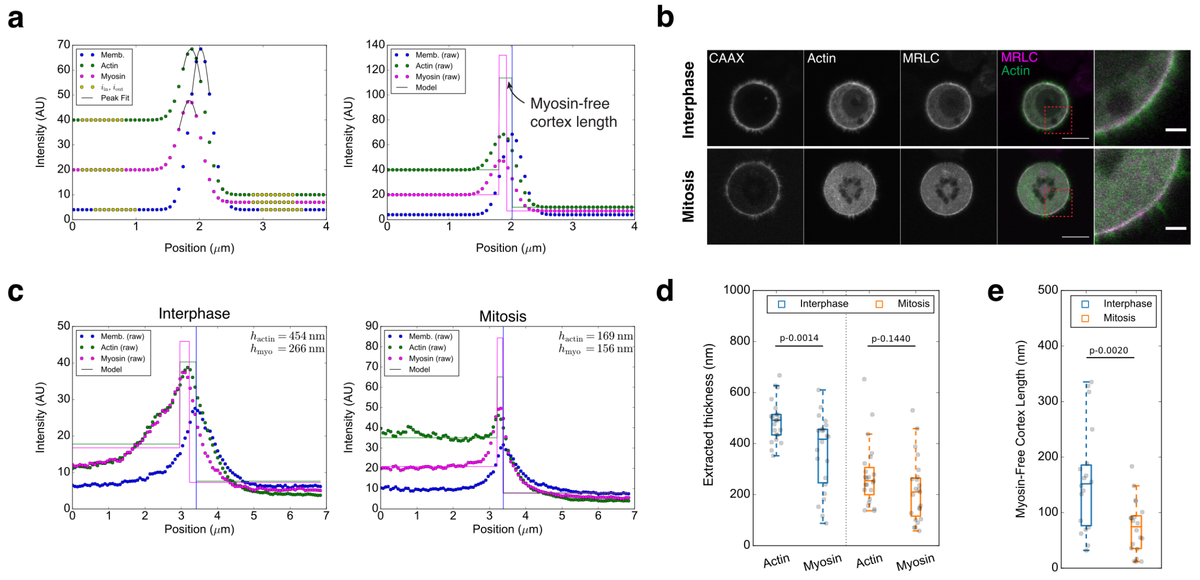

Supplementary Fig. 3: Cortical actin and myosin thickness assessed by confocal microscopy. **a,** Left: simulated linescans of cortical actin and myosin and the plasma membrane, based on a simple model of the cortex, where the actin and myosin layers are modeled as a sum of step functions convolved with the PSF of the microscope used for cell imaging (see Methods for details). A number of parameters are extracted from linescans, including intracellular background (i_{in}) and extracellular background (i_{out}) and the intensity and position of the intensity peak. Right: The parameters extracted from the linescans are fit to the model of the cortex. Fitting the simulated linescan data (circles) yields the mean position of the plasma membrane and the thickness and intensities of the actin and myosin layers (solid lines). As the model assumes the cytoplasmic edges of the actin and myosin cortical layers overlap, the thickness of the myosin layer is equivalent to the myosin penetration depth into the actin layer, and the difference between the actin thickness and myosin thickness represents the myosin-free cortex length. **b,** Representative equatorial section images of MBCD-treated live suspension S-HeLa cells blocked in interphase or mitosis and expressing BFP-CAAX (plasma membrane marker), GFP-Actin and Myosin Regulatory Light Chain (MRLC)-mCherry. Scale bars, 10 μm. **c,** Cortical linescans and analysis corresponding to the images in **b**. Dots: cortical intensities from the linescans. Solid lines: underlying model distributions of actin and myosin extracted as highlighted in **a**. **d, e** Extracted thicknesses of actin and myosin cortical layers **d**, and resulting myosin-free cortex length in interphase and mitotic cells **e** extracted from linescans as highlighted in **c**. Boxes in all box plots extend from the 25th to 75th percentiles, with a line at the median. Whiskers extend to ×1.5 the interquartile range or the max/min data points if they fall within 1.5 times the interquartile range. Each dot on the box plots represents a measurement from a single cell. Statistics: Welch t-test (two-tailed).

Supplementary Fig. 4

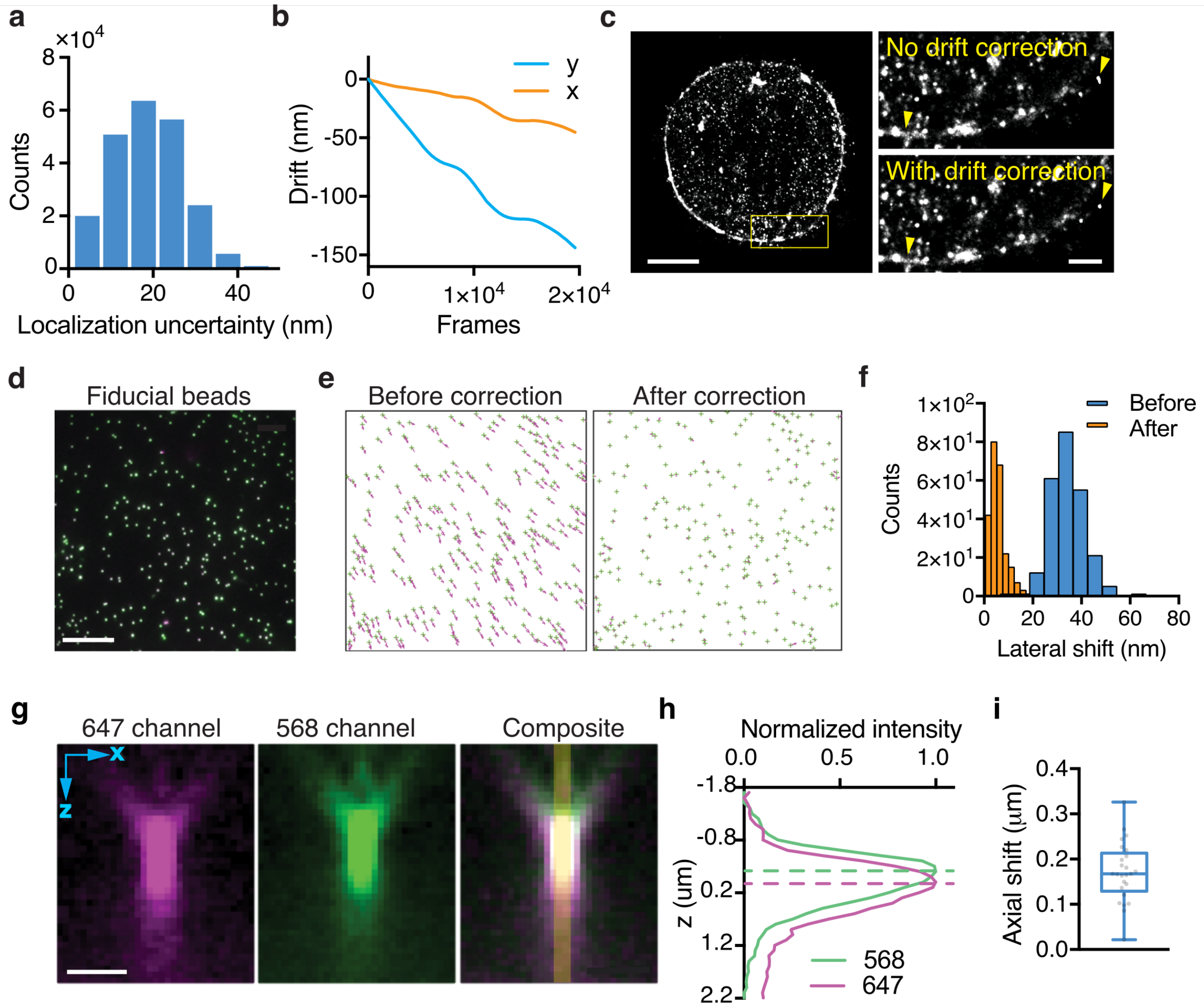

Supplementary Fig. 4: Performance evaluation of dSTORM imaging. a, Representative histogram of localization uncertainties estimated from ~ 1.4×10^6 localized molecules from one dSTORM acquisition (thresholded at 50 nm). **b,** Typical lateral drift experienced during a dSTORM acquisition, measured by tracking fiducial beads. **c,** A dSTORM image of myosin with and without lateral drift correction. Arrows highlight bead features effectively aligned by drift correction. Scale bars, 5 µm (leftmost image) and 0.5 µm (highlighted ROI). **d-f,** Measurement of the chromatic shift between 568 and 647 channels. **d,** Representative image of fiducial multicolor fluorescent beads imaged in both the 568 and 647 (depicted green and magenta respectively) channels. Scale bar, 10 µm. **e,** Representative arrow plots and **f,** histograms of chromatic shift before and after correction. Arrows connect the same bead as imaged in the two channels and were scaled up 20 times for visualization purposes. **g-k**, Measurement of the axial shift between the 568 and 647 channels. **g,** Axial profile of the experimental PSF in 568 and 647 (depicted green and magenta respectively) channels, reconstructed from a 4 µm z-stack of a multicolor fluorescent bead. Scale bar, 1µm. **h,** Normalized intensity profiles of the imaged PSF (for both the 568 and 647 channels) along the axial axis in the region highlighted in yellow in **g,** from which the axial shift was determined **i,** n = 25 beads. Boxplot shows 25th to 75th percentiles, the median, and whiskers from minimum to maximum.

Supplementary Fig. 5

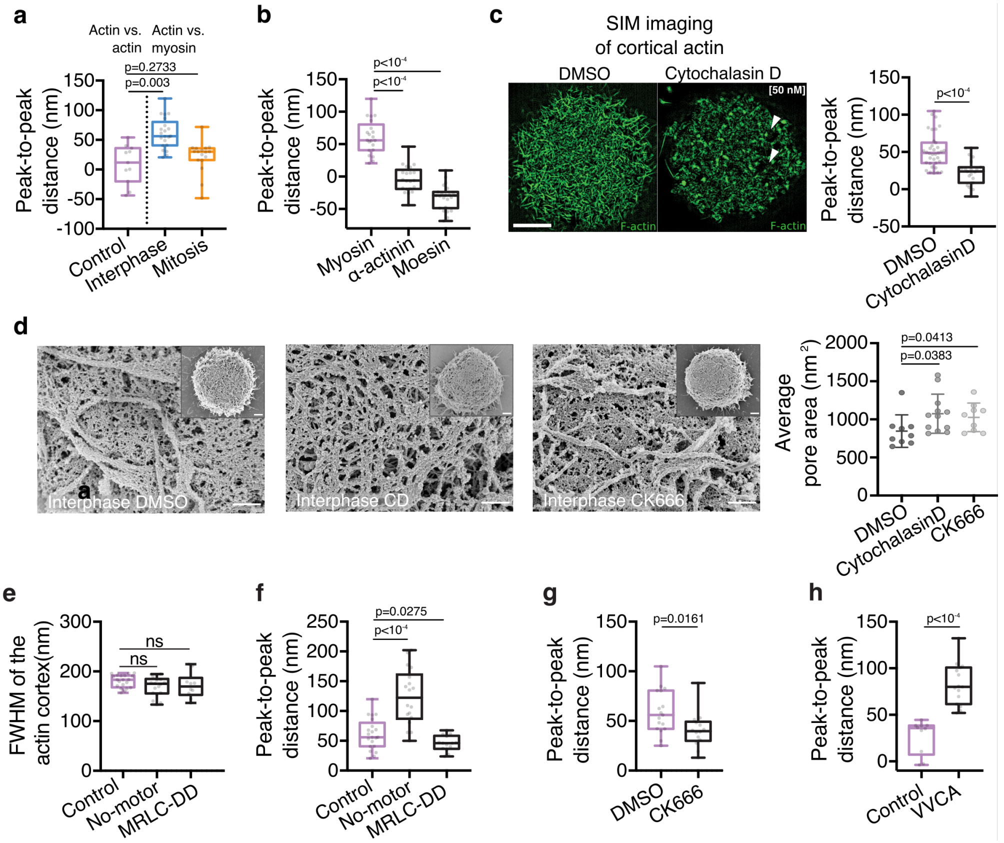

Supplementary Fig. 5: Additional measurements on cortical intensity profiles from dSTORM images. a, Distance between the peaks of the intensity profiles ('Peak-to-peak distance') of cortical actin labeled in 2 different colors (control, n=11 cells), and of cortical actin and myosin in in rounded interphase and mitotic cells (n=19 and 16 cells, respectively); 3 independent experiments for each condition. Boxplots show 25th to 75th percentiles, the median, and whiskers from minimum to maximum. **b,** Peak-to-peak distance of cortical actin and myosin (control), and cortical actin and α-actinin or moesin, in rounded interphase cells; n = 19, 23 and 21 cells, respectively; at least 4 independent experiments. Boxplots show 25th to 75th percentiles, the median, and whiskers from minimum to maximum. **c,** Left: Representative SIM images of the actin cortex in rounded HeLa cells treated with DMSO (control) or 50 nM CD. Arrowheads highlight apparent holes in the actin cortex. Scale bar, 5 µm. Right: peak-to-peak distance of cortical actin and myosin in rounded interphase HeLa cells treated with DMSO (control) or 50 nM CD, n = 35 and 19 cells, respectively; at least 3 independent experiments. Boxplots show 25th to 75th percentiles, the median, and whiskers from minimum to maximum. **d,** SEM imaging of the outer surface of the interphase cortex, corresponding to conditions in **Figure 3b, d,** and quantification of the average actin pore area; (DMSO n=9 cells, CD n=12 cells, CK666 n=9 cells; three independent experiments). Scatter plot with median with the interquartile range. **e,f** Peak-to-peak distance of cortical actin and myosin **(e)** and actin cortex thickness as assessed by FWHM **(f)** in HeLa cells expressing MRLC-GFP (control), ΔN592 NMHC IIA-GFP or EGFP-MRLC2-DD myosin mutants. n = 19, 15, 14 cells, respectively; 3 independent experiments. Boxplots show 25th to 75th percentiles, the median, and whiskers from minimum to maximum**. g,** Peak-to-peak distance of cortical actin and myosin rounded interphase HeLa cells treated with DMSO (control) or 20 µM CK666; n = 17, 14 cells, respectively; 3 independent experiments. Boxplots show 25th to 75th percentiles, the median,

and whiskers from minimum to maximum**. h,** Peak-to-peak distance of cortical actin and myosin in mitotic HeLa cells expressing an empty BFP plasmid (control) or VVCA-BFP; n = 10, 15 cells in 2 and 3 independent experiments respectively. Boxplots show 25th to 75th percentiles, the median, and whiskers from minimum to maximum. Statistics: Mann-Whitney (two-tailed) or Welch's t-test (two-tailed).

Supplementary Fig. 6

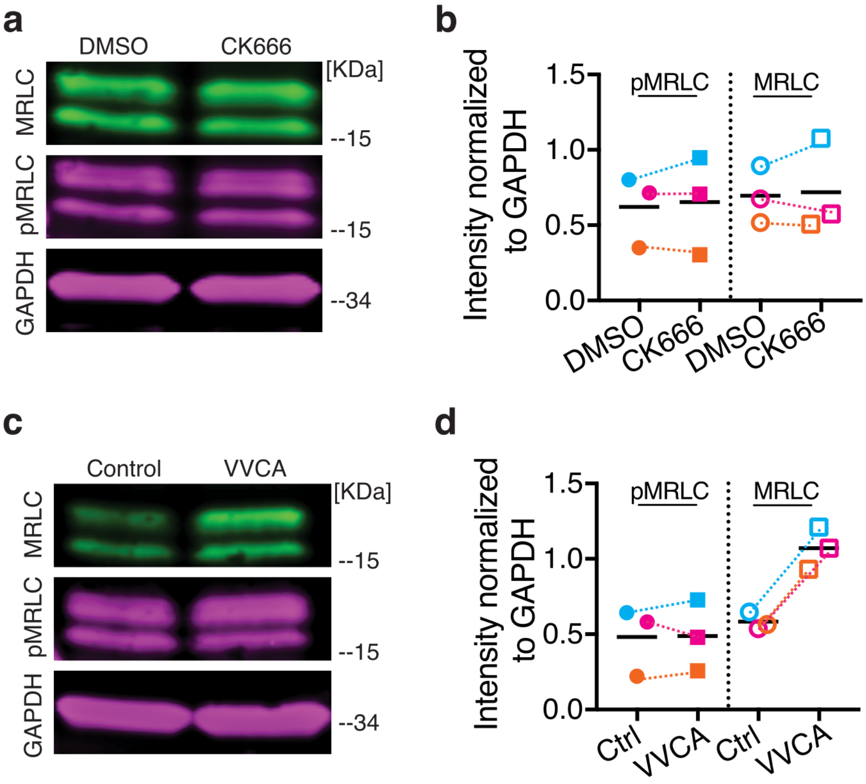

Supplementary Fig. 6: Myosin levels upon actin perturbation. a, c, Fluorescent immunoblot of MRLC (green), pMRLC (Ser19, magenta) and loading control GAPDH (magenta) for interphase HeLa cells treated with DMSO (control) or 20 µM CK666 **a;** or overexpressing an empty BFP plasmid (control) or VVCA-BFP, **c**. Uncropped blots are shown in Fig. S7. **b, d,** Corresponding quantifications: normalized intensities of pMRLC and MRLC in DMSO (circles) and CK666 (squares) treated cells **b;** or cells transfected with empty BFP (circles) and VVCA-BFP (squares). Data points from individual experiments connected by dotted lines.

Supplementary Fig. 7

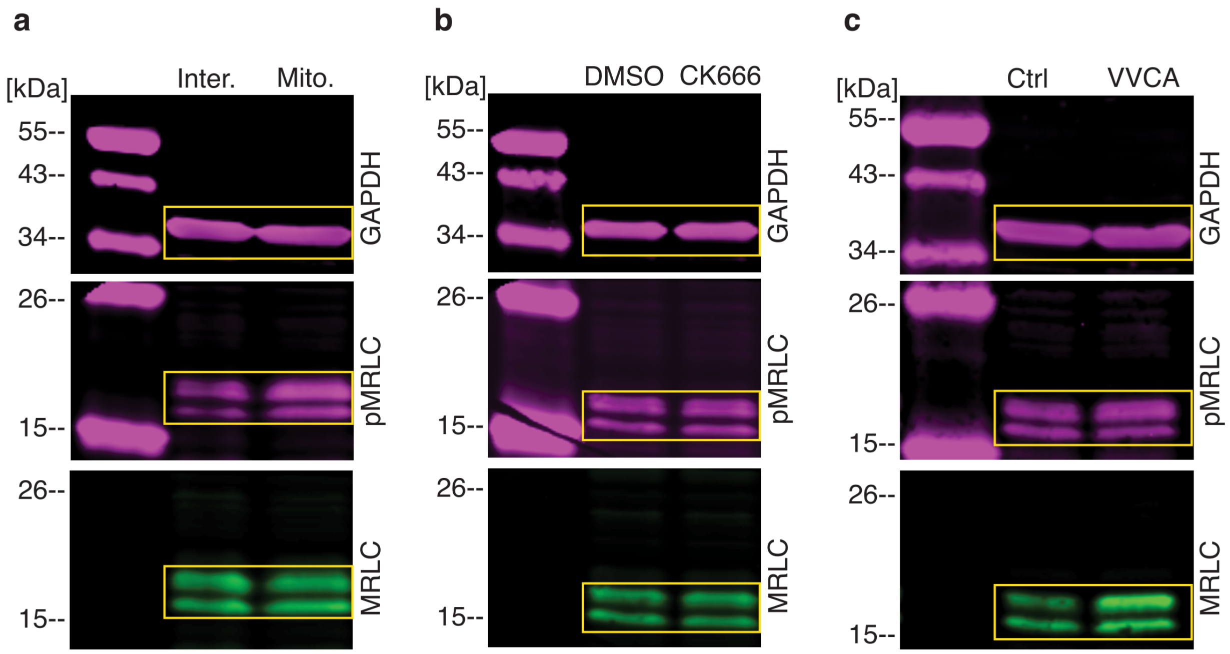

Supplementary Fig. 7: Uncropped Western blots. Uncropped fluorescent immunoblots corresponding to **a**, Supplementary Fig. 1f, **b,** Supplementary Fig. 6a and **c,** Supplementary Fig. 6c.
