# Supplementary material for: Extent of myosin penetration within the actin cortex regulates cell surface mechanics
Source: Nat Commun. 2021 Nov 11;12:6511. doi: 10.1038/s41467-021-26611-2 (PMC8586027; doi:10.1038/s41467-021-26611-2)
Supplement: Supplementary file 2 — Reporting Summary [file 41467_2021_26611_MOESM2_ESM.pdf]

nature research

Corresponding author(s): Ewa. K. Paluch

Last updated by author(s): Jul 20, 2021

Reporting Summary

Nature Research wishes to improve the reproducibility of the work that we publish. This form provides structure for consistency and transparency in reporting. For further information on Nature Research policies, see our Editorial Policies and the Editorial Policy Checklist.

Statistics

For all statistical analyses, confirm that the following items are present in the figure legend, table legend, main text, or Methods section.

n/a	Confirmed	
☐	☒	The exact sample size (*n*) for each experimental group/condition, given as a discrete number and unit of measurement
☐	☒	A statement on whether measurements were taken from distinct samples or whether the same sample was measured repeatedly
☐	☒	The statistical test(s) used AND whether they are one- or two-sided<br>*Only common tests should be described solely by name; describe more complex techniques in the Methods section.*
☒	☐	A description of all covariates tested
☐	☒	A description of any assumptions or corrections, such as tests of normality and adjustment for multiple comparisons
☐	☒	A full description of the statistical parameters including central tendency (e.g. means) or other basic estimates (e.g. regression coefficient) AND variation (e.g. standard deviation) or associated estimates of uncertainty (e.g. confidence intervals)
☐	☒	For null hypothesis testing, the test statistic (e.g. *F*, *t*, *r*) with confidence intervals, effect sizes, degrees of freedom and *P* value noted<br>*Give P values as exact values whenever suitable.*
☒	☐	For Bayesian analysis, information on the choice of priors and Markov chain Monte Carlo settings
☒	☐	For hierarchical and complex designs, identification of the appropriate level for tests and full reporting of outcomes
☒	☐	Estimates of effect sizes (e.g. Cohen's *d*, Pearson's *r*), indicating how they were calculated

Our web collection on statistics for biologists contains articles on many of the points above.

Software and code

Policy information about availability of computer code

Data collection	The following commercial software(s) were used: Zeiss ZEN software (Elyra 7 microscope, no version number from developer), Leica LAS X software (Leica TCS SP8 STED microscope, no version number from developer), JPK software (compatible with JPK CellHesion module for AFM measurements, no version number from developer).
Data analysis	The following open-source software(s) were used: ThunderSTORM plug-in to ImageJ/Fiji (Version 2.1.0), Image Studio Lite software (no version number from developer), Python (Version 3.8).<br>The following commercial software(s) were used: MATLAB (R2018b), Graphpad Prism (Version 9.0.2), Zeiss ZEN Black (no version number from developer).<br>Custom MATLAB code is available on GitHub with user guide at https://github.com/PaluchLabUCL, as detailed in the code availability section of the manuscript.

For manuscripts utilizing custom algorithms or software that are central to the research but not yet described in published literature, software must be made available to editors and reviewers. We strongly encourage code deposition in a community repository (e.g. GitHub). See the Nature Research guidelines for submitting code & software for further information.

Data

Policy information about availability of data

All manuscripts must include a data availability statement. This statement should provide the following information, where applicable:

- Accession codes, unique identifiers, or web links for publicly available datasets
- A list of figures that have associated raw data
- A description of any restrictions on data availability

All quantified data is available in the Main Text or in the Supplementary Information file; each box-plot shows both the data distribution and the individual data

points. The raw data generated in this study underlying Fig. 1b and Fig.4d is provided in the Source Data file. Source data are provided with this paper. The raw datasets generated during the study are not publicly available owing to their large size, but are available from the corresponding author upon reasonable request.

Field-specific reporting

Please select the one below that is the best fit for your research. If you are not sure, read the appropriate sections before making your selection.

- [x] Life sciences
- [ ] Behavioural & social sciences
- [ ] Ecological, evolutionary & environmental sciences

For a reference copy of the document with all sections, see nature.com/documents/nr-reporting-summary-flat.pdf

Life sciences study design

All studies must disclose on these points even when the disclosure is negative.

Sample size	Sample sizes denoted n refer to n cells, from independent biological replicate experiments as specifically stated in all figure legends. All boxplots are defined in terms of minima, maxima, centre, bounds of box and whiskers and percentile in the figure legends, with all data points displayed. No statistical method was used to predetermine sample size. We instead set a target sample size for sufficient numbers of cells (>10) to ensure a normal distribution around the mean. Sample size was limited only by throughput, each experiment was repeated multiple times on independent days and pooled together for analysis. For method demonstration images and data, (Supp. Fig. 4 for example) no statistics were computed. All figure legends include the statistical test used.
Data exclusions	No data was excluded from this study.
Replication	For STORM experiments, a calibration image was acquired at each day of independent experiments. For AFM studies, the cantilever sensitivity was calibrated by acquiring a force curve on a glass coverslip prior to imaging cells. Replications of experiments were successful, and biological replicate conditions are clearly stated in the figure legends.
Randomization	To categorize images into either interphase or mitotic cells, a DAPI staining was used to visually identify the stage in the cell cycle. Otherwise, groups were allocated by experimental condition, for example unperturbed vs. drug-treated cells. No sub-sampling took place, such that randomization was not required.
Blinding	Blinding was not possible since experimental conditions were evident from each imaging data set.

Reporting for specific materials, systems and methods

We require information from authors about some types of materials, experimental systems and methods used in many studies. Here, indicate whether each material, system or method listed is relevant to your study. If you are not sure if a list item applies to your research, read the appropriate section before selecting a response.

Materials & experimental systems

n/a	Involved in the study	
[ ]	[x]	Antibodies
[ ]	[x]	Eukaryotic cell lines
[x]	[ ]	Palaeontology and archaeology
[x]	[ ]	Animals and other organisms
[x]	[ ]	Human research participants
[x]	[ ]	Clinical data
[x]	[ ]	Dual use research of concern

Methods

n/a	Involved in the study	
[x]	[ ]	ChIP-seq
[x]	[ ]	Flow cytometry
[x]	[ ]	MRI-based neuroimaging

Antibodies

Antibodies used

The following primary antibodies were used:
anti-phospho-MRLC2 (Ser19) (rabbit, #3671S, Cell Signaling Technology)
anti-phospho-MRLC2 (Thr18/Ser19) (rabbit, #3674, Cell Signaling Technology)
anti-phospho-MRLC (Ser 19) (mouse,#3675, Cell Signaling Technology)
anti-MRLC (rabbit, #3672, Cell Signaling Technology)
anti-GAPDH (clone 1D4, mouse, NB300-221, Novus Biologicals)
anti-MRLC (mouse, M4401, Sigma)
anti-MYH9 (rabbit, #14971001, Covance)
anti-MYH10 (rabbit, #14942501, Covance)

The following secondary antibodies/nanobodies were used:
donkey-anti-mouse Alexa Fluor 488 (A-21202, Invitrogen)
donkey-anti-rabbit Alexa Fluor 488 (A-21206, Invitrogen)

	Alexa Fluor 647 anti-GFP nanobody (gb2AF647, Chromotek)<br>goat-anti-mouse Alexa Fluor 680 (A-21058 ThermoFisher)<br>goat-anti-rabbit Alexa Fluor 790 (A11369, ThermoFisher).<br>Dilutions and incubation timing is detailed in the Methods section.
Validation	All primary antibodies were purchased from companies and the corresponding western blot figures, immunofluorescence images, conditions and concentrations of use were available on the manufacturers website. We would kindly refer to the manufacturers website and corresponding data sheets to find statements of validation and citations. The expected intracellular localization of each antibody further validated the antibodies used for immunofluorescence. We also provide example publications for the primary antibodies listed below, as listed on the website of the manufacturer:<br><br>anti-phospho-MRLC2 (Ser19) (rabbit, #3671S, Cell Signaling Technology)<br>(I. Yanakieva et al., 2019. J Cell Biol. 218(10)3272-3289)<br><br>anti-phospho-MRLC2 (Thr18/Ser19) (rabbit, #3674, Cell Signaling Technology)<br>(Y. Huang et al.,2019. Cells. 8(10):1264)<br><br>anti-phospho-MRLC (Ser 19) (mouse,#3675, Cell Signaling Technology)<br>(S. Ito et al., 2017. Nat Comm. 8(1):1834)<br><br>anti-MRLC (rabbit, #3672, Cell Signaling Technology)<br>(D. E. Mason et al.,2019. J C Biol. 218(4):1369-1389)<br><br>anti-GAPDH (clone 1D4, mouse, NB300-221, Novus Biologicals)<br>(L. Xiong et al., 2020. PLoS Biol. 28(6):e3000731)<br><br>anti-MRLC (mouse, M4401, Sigma)<br>(B. Guvenc et al., 2010. FEBS Lett. 584(13):2862-6)<br><br>anti-MYH9 (rabbit, #14971001, Covance) - no longer available.<br>anti-MYH10 (rabbit, #14942501, Covance) - no longer available.

Eukaryotic cell lines

Policy information about cell lines

Cell line source(s)	HeLa TDS cells were gifted from MPI-CBG Technology Development Studio (TDS, Dresden, Germany). S-HeLa cell line was derived from HeLa TDS by constant culturing on low-adherent flasks (Corning). Stated in Line 1 of Methods section.
Authentication	Cell lines used were not authenticated.
Mycoplasma contamination	All cell lines tested negative for mycoplasma contamination (stated in Methods section), and were regularly tested (every 2 weeks) by a person other than the keeper.
Commonly misidentified lines<br>(See ICLAC register)	In this study HeLa TDS cells were used. These lines have not been flagged as commonly misidentified by ICLAC or NCBI Biosample.
